# HSP90-dependent PUS7 overexpression facilitates the metastasis of colorectal cancer cells by regulating LASP1 abundance

**DOI:** 10.1186/s13046-021-01951-5

**Published:** 2021-05-14

**Authors:** Dan Song, Ming Guo, Shuai Xu, Xiaotian Song, Bin Bai, Zhengyan Li, Jie Chen, Yanxin An, Yongzhan Nie, Kaichun Wu, Shiqi Wang, Qingchuan Zhao

**Affiliations:** 1 State key Laboratory of Cancer Biology, National Clinical Research Center for Digestive Diseases and Xijing Hospital of Digestive Diseases, Fourth Military Medical University, 710032 Xi’an, Shaanxi Province China; 2 Department of Anesthesiology, Renmin Hospital of Wuhan University, Wuhan, 430060 Hubei Province China; 3 Department of General Surgery, Center for Minimally Invasive Gastrointestinal Surgery, Southwest Hospital, Third Military Medical University, No. 30 Gao Tan Yan Road, Chongqing, 400038 China; 4 Department of General Surgery, the First Affiliated Hospital of Xi ’an Medical University, No. 48 Fenghao West Road, Lianhu District, Xi’an, 710077 Shaanxi Province China; 5 Department of Gastrointestinal Surgery, Xijing Hospital of Digestive Diseases, Xijing Hospital, Fourth Military Medical University, Xi’an, 710032 Shaanxi Province China

**Keywords:** Colorectal cancer, Metastasis, PUS7, HSP90, LASP1

## Abstract

**Background:**

Pseudouridine synthase (PUS) 7 is a member of the PUS family that catalyses pseudouridine formation. It has been shown to be involved in intellectual development and haematological malignancies. Nevertheless, the role and the underlying molecular mechanisms of PUS7 in solid tumours, such as colorectal cancer (CRC), remain unexplored. This study elucidated, for the first time, the role of PUS7 in CRC cell metastasis and the underlying mechanisms.

**Methods:**

We conducted immunohistochemistry, qPCR, and western blotting to quantify the expression of PUS7 in CRC tissues as well as cell lines. Besides, diverse in vivo and in vitro functional tests were employed to establish the function of PUS7 in CRC. RNA-seq and proteome profiling analysis were also applied to identify the targets of PUS7. PUS7-interacting proteins were further uncovered using immunoprecipitation and mass spectrometry.

**Results:**

Overexpression of PUS7 was observed in CRC tissues and was linked to advanced clinical stages and shorter overall survival. *PUS7* silencing effectively repressed CRC cell metastasis, while its upregulation promoted metastasis, independently of the PUS7 catalytic activity. LASP1 was identified as a downstream effector of PUS7. Forced *LASP1* expression abolished the metastasis suppression triggered by *PUS7* silencing. Furthermore, HSP90 was identified as a client protein of PUS7, associated with the increased PUS7 abundance in CRC. NMS-E973, a specific HSP90 inhibitor, also showed higher anti-metastatic activity when combined with PUS7 repression. Importantly, in line with these results, in human CRC tissues, the expression of PUS7 was positively linked to the expression of HSP90 and LASP1, and patients co-expressing HSP90/PUS7/LASP1 showed a worse prognosis.

**Conclusions:**

The HSP90-dependent PUS7 upregulation promotes CRC cell metastasis via the regulation of LASP1. Thus, targeting the HSP90/PUS7/LASP1 axis may be a novel approach for the treatment of CRC.

**Supplementary Information:**

The online version contains supplementary material available at 10.1186/s13046-021-01951-5.

## Background

Colorectal cancer (CRC) is a primary contributor to cancer-related deaths globally [1]. Metastasis constitutes the major factor contributing to the high relapse rate and poor survival among individuals with CRC [2–4]. Despite advancements in both diagnosis and systemic treatment, patients with advanced distal organ metastasis still exhibit a poor prognosis [3]. In the past decade, immense progress has been made in the context of research and development. However, the molecular mechanisms underlying CRC metastasis are still unclear, and advanced metastatic CRC remains incurable [3, 5]. Thus, the mechanisms underlying CRC metastasis should be explored for the development of specific strategies for patients with metastatic CRC.

Pseudouridine synthases (PUSs) comprise a class of proteins within the following six families: TruA, TruB, TruD, RsuA, RluA, and PUS10p. They catalyse the formation of pseudouridine (Ψ) mainly in an RNA-independent manner [6]. These enzymes have been associated with human diseases and tumorigenesis. For instance, the PUS1-mediated pseudouridylation participates in NR signal transduction in breast cancer cells [7]. PUS3 homozygous variants are associated with intellectual disability [8]. PUS10 is a risk locus for Crohn’s disease [9] and lung cancer [10], and is required for TRAIL-induced apoptosis in prostate cancer [11]. Of note, some functions of PUSs are independent of their enzymatic activity. For example, bacterial TruB acts as a tRNA-folding chaperone independent of catalysis [12]. Additionally, catalytic null PUS10 plays a role in miRNA biogenesis [13]. Collectively, these studies highlight the importance of PUSs in biological processes and the possibility of additional non-catalytic PUSs functions in tumour development.

Our understanding of the activity and function of PUSs remains incomplete. Most studies of PUSs have focused on the identification of pseudouridylation sites; whether PUSs impact cellular phenotypes including tumour growth or metastasis as well as the mechanisms underlying PUSs dysregulation are yet unknown. Taking PUS7 as an example (the only member of the TruD family) [14–17], increasing pieces of evidence indicate that the PUS7-triggered Ψ modification participates in numerous biological processes, such as embryonic stem cell self-renewal, fate determination, and intellectual development [18, 19]. Moreover, PUS7 has been reported to modify MALAT1, a tumour-related and well-studied long non-coding RNA [15, 20]. Additionally, Guzzi et al. reported that the PUS7-initiated Ψ modifications in tRNA-derived small fragments affect translation and tumorigenesis [21]. Of note, our pre-tests indicated that *PUS7* is specifically highly expressed in CRC tissues and may promote CRC cells metastasis, supporting the hypothesis of a correlation between aberrant PUS7 expression and cancer development. However, except for the involvement of PUS7 in human haematological malignancies, little is known about the cellular functions of PUS7 in other solid tumours.

Although, the molecular mechanisms of tumour metastasis are diverse and complex, cell detachment, migration and infiltration are decisive steps during tumour dissemination [3]. Several transcription factors and cytoskeletal proteins serve as key contributors to these processes [22]. Among the genes regulated by PUS7, LIM and SH3 protein 1 (*LASP1*) has been reported as a complex nuclear transcriptional regulator [23], responsible for the reorganization of the cytoskeleton, and consequently, for cellular movement [24]. Moreover, previous studies have proposed that *LASP1* is an oncogene; it can promote tumorigenesis and has a great prognosis value in variety of cancer patients [25–33]. In fact, in CRC, LASP1 was identified as a metastasis-related protein, important for the TGF-β-triggered EMT process [34], hippo signalling pathway [35], and interaction with N-WASP [36] or COPS5 [29] to trigger the migration and infiltration of CRC cells.

Previous studies revealed that PUS7 is regulated by heat shock or other stress conditions [16, 37], via ubiquitination or succinylation [38–40]. Thus, we reasoned that heat shock proteins (HSPs) may regulate PUS7 and assist its post-translational modifications. HSPs have been reported to impact the folding, maturation, and degradation of client oncogenic proteins, participating in the modulation of the proliferation, apoptosis, and metastasis in the context of human cancers [41]. Furthermore, HSPs-related small-molecule inhibitors were proposed as promising options for the treatment of CRC [42–44]. Although early-phase clinical studies revealed that monotherapy had limited clinical efficacy, they showed that the combination of HSPs inhibitors with targeted agents improves their antitumor activity [45].

Here, we discovered a remarkable upregulation of PUS7 in CRC tissues; as far as we are concerned, this is the first time such results are reported. Additionally, the overexpression of PUS7 was linked to a poor prognosis. Importantly, we show that PUS7 promotes metastasis in the context of CRC via the modulation of LASP1 in an RNA pseudouridylation-independent manner. Moreover, HSP90 was found to be a novel binding partner of PUS7. In fact, we found that HSP90 is behind the increase in the PUS7 protein levels in CRC, promoting protein stability and decreasing the proteasomal degradation of PUS7. Importantly, *PUS7* knockdown partially reduced the HSP90-enhanced CRC infiltration as well as metastasis. Additionally, NMS-E973, a distinct HSP90 suppressor, showed greater anti-metastatic activity when combined with *PUS7* repression. Of note, the expression of HSP90 was positively linked to the expression of PUS7 and LASP1 in CRC tissues, and individuals with the co-expression of HSP90/PUS7/LASP1 showed a worse prognosis. Altogether, our results suggest that the HSP90/PUS7/LASP1 axis may be a promising target for the treatment of metastatic CRC.

## Methods

### Cell culture

Human CRC cells (HIEC-6, DLD-1, RKO, HCT-8, HCT-116, SW480, and SW620 cells) were stored in our laboratory and authenticated using STR profiling. The HIEC-6, DLD-1, RKO, HCT-8, HCT-116, and SW620 cells were inoculated into Gibco-RPMI 1640 medium (Grand Island, NY, USA) enriched with 10% Gibco-FBS, 100 μg/mL Gibco-penicillin, and 100 μg/mL Gibco-streptomycin under 5% CO_2_ and 37 °C conditions. The SW480 cell lines were inoculated into L15 medium (HyClone; Logan, Utah, USA) enriched with 10% Gibco-FBS, 100 μg/mL Gibco-penicillin, and 100 μg/mL Gibco-streptomycin and incubated at 37 °C.

### Tumour samples

Fresh CRC and surrounding tissue specimens were obtained from subjects who had undergone surgery for CRC at the Xijing Hospital of Digestive Diseases. The specimens were snap-frozen immediately in nitrogen. All the specimens were pathologically and clinically labelled. Haematoxylin-eosin staining conducted at the Department of Pathology of Xijing Hospital was employed to confirm the histomorphology of all the primary tumour samples and the regional lymph nodes. The Xijing Hospital’s Protection of Human Subjects Committee approved the study. All participants provided informed consent. A detailed description of the patient samples acquired from clinical datasets is provided in the supplementary Table S5.

### qPCR

The TaKaRa MiniBEST Universal RNA Extraction Kit (Cat# 9767, Takara, Japan) was employed to isolate total RNA, and then cDNA was synthesised from the total RNA via reverse transcription using the Takara-reverse transcription kit (Cat# RR036A, Japan) as described by the manufacturer. Thereafter, the Takara-SYBR Green PCR Kit (Cat# RR820A, Japan) was employed to perform qPCR on the Bio-Rad CFX96 system (CA, USA) using the following program: pre-denaturation at 95 °C for 10 min, 40 cycles of denaturation at 95 °C for 15 s, primer annealing at 60 °C for 1 min, and final elongation at 72 °C for 30 s. The generated melting curve was evaluated, and Applied Biosystems-SDS 1.9.1 software was used to determine the Ct values during the exponential amplification phase. The reactions were performed in triplicate. The sequences of the primers are indicated in Supplementary Table S1.

### WB analysis

The RIPA buffer enriched with a cocktail of Millipore protease inhibitor and phosphatase inhibitor was employed to prepare whole cell lysates. Protein concentration was determined using the Thermo Fisher Scientific BCA Protein Assay Kit. Afterwards, protein fractionation was performed using SDS-PAGE gels and then the proteins were blotted onto nitrocellulose membranes. Subsequently, 5% dry milk in TBST (0.05% Tween 20, 120 mM Tris– HCl (pH 7.4), and 150 mM NaCl) was employed to block nonspecific binding for 1 h at room temperature (25 °C). The membranes were incubated with the specified primary antibodies overnight at 4 °C. Antibodies against HSP90 (Abcam, USA, cat#ab2928, 1:2000), PUS7 (Abcam, USA, cat#ab224119, 1:1000), LASP1 (Proteintech, Wuhan, China, cat#10515–1-AP, 1:10000), the loading control β-actin (Sigma, cat#A1978, 1:4000), tubulin (Proteintech, Wuhan, China, cat#11224–1-AP, 1:20000), and GAPDH (Proteintech, Wuhan, China, cat#10494–1-AP, 1:10000) were used. Afterwards, we rinsed the membranes thrice with TBST and inoculated them with an HRP-labelled secondary antibody. The protein bands were visualised using Bio-Rad ChemiDoc XRS+ Imaging System and quantified with Image Lab (Bio-Rad).

### IHC

Slides (4-μm-thick) were placed in an incubator for 1 h at 60 °C and then deparaffinised in xylene, followed by dehydration with graded ethanol. Subsequently, antigen retrieval was performed using boiling citrate buffer (0.01 M, pH 6.0) for 2 min. After rinsing in PBS (pH 7.5) three times for 5 min each, we immersed the slides in a 3% hydrogen peroxide solution in methanol for 10 min to repress endogenous peroxidase activity, and then pre-incubated them with 5% goat serum for 30 min at room temperature (25 °C) to block nonspecific binding. Thereafter, the slides were incubated with the specified primary antibody at 4 °C in blocking buffer overnight. Antibodies against HSP90 (Abcam, USA, cat#ab2928, 1:5000), PUS7 (Abcam, USA, cat#ab224119, 1:3200), LASP1 (Proteintech, Wuhan, China, cat#10515–1-AP, 1:3000), and pseudouridine mAb (MBL, Nagoya, Japan, CODE No. D347–3, 1 μg/mL) were used. The slides were further rinsed with PBS and then inoculated with the peroxidase-labelled polymer conjugated to goat anti-rabbit/mouse immunoglobulin for 30 min at room temperature (25 °C). Diaminobenzidine tetrahydrochloride was then employed to visualize immunostaining, followed by counter-staining of the sections with haematoxylin. Dehydration was performed, and the slides were covered with coverslips.

Results of the immunohistochemistry staining assay were analysed with a score index considering the intensity of staining and the percentage of tumour cells with an unequivocal positive reaction. Brown signals in cells indicated positive reactions. A staining index (values between 0 and 12) was established as a product of the score of staining intensity and the score of positive proportion. The intensity was scored as follows: 0 (negative), 1 (weak), 2 (medium), or 3 (strong). The proportion of positive cells was scored as follows: 0 (negative), 1 (1–25%), 2 (26–50%), 3 (51–75%), or 4 (76–100%). All IHC scores were examined independently by two pathologists without prior knowledge of patient data. The differences in scores were discussed by the two pathologists, and a consensus was reached. A third pathologist participated in the discussion when necessary.

### Construction of lentivirus and stable cell lines

Overexpression and knockdown lentiviral vectors were designed and provided by Genechem Co. Ltd. (China). The *PUS7* and *LASP1* knockdown shRNA sequences were as follows: *PUS7* shRNA: cgTTGCATTGATAGGCCCATT and *LASP1* shRNA: ctGGATAAGTTCTGGCATAAA. Cells were transfected at an MOI of 30–50. At 72 h after infection, the cells were selected for 2 weeks using 1–4 μg/mL puromycin. We used the selected pools of cells for subsequent assays. Rescued cell lines were obtained via knockdown or overexpression of the downstream proteins subsequent to the establishment of the stable cell lines transfected with lentiviral vectors targeting the upstream factors.

### In vitro migration and invasion assays

An 8-μm pore 24-well Corning Transwell plate (MA, USA) was employed to assay the migration and invasion along with the potential of all cell lines. In the invasion analysis, we coated the chamber inserts containing an 8-μm pore size with 200 mg/mL Matrigel (Corning), and the upper compartment was inoculated with 6 × 10^4^ cells suspended in serum-free media, and 20% FBS was employed as a chemo-attractant and applied to the lower chamber. In the cell migration assays, the upper compartment containing the non-coated membrane was inoculated with 6 × 10^4^ cells. The cells were allowed to grow at 37 °C for a specified time, followed by fixation with 4% paraformaldehyde. Afterwards, crystal violet-staining was performed, and then we counted the cells at × 100 under a microscope (Olympus, Tokyo, Japan). All assays were performed in triplicate. The degree of invasion and migration was determined as a ratio of the number of treated cells to control cells.

### In vivo metastatic model

Six-week-old BALB/C nude mice were maintained according to our institution’s protocols for ethical animal care. The Committee of the Use of Live Animals in Teaching and Research (CULATR) of the Fourth Military Medical University approved all animal experiments. We injected 1 × 10^6^ cell suspensions in 150 μL PBS into the tail vein in each group of mice for in vivo metastasis assays. In the HSP90 inhibitor experiment, mice were administered 10 mg/kg NMS-E973 (Selleck, S7282, Houston, TX, USA) intraperitoneally every 2 days after 2 weeks of CRC cell transplantation. Eight weeks post-injection, the mice were sacrificed, and lungs were dissected for subsequent histological assessment.

### Immunofluorescence

Cells (4 × 10^5^ cells/well) were inoculated onto a Millipore Millicell EZ SLIDE and incubated overnight. Subsequently, the slides were rinsed thrice with PBS, followed by fixation with 4% paraformaldehyde for 30 min. Another PBS wash of the cells was performed for 3 min, after which the slides were treated with 5% BSA for 30 min. Afterwards, the slides were incubated with primary antibodies in blocking buffer overnight. The primary antibodies were mouse anti-human PUS7 antibody (GeneTex, CA, USA, Cat No. GTX83757, 1:100), rabbit anti-human HSP90 antibody (Abcam, USA, cat#ab2928, 1:200), and rabbit anti-human LASP1 antibody (Proteintech, Wuhan, China, cat#10515–1-AP, 1:100). Then, the slides were rinsed thrice with PBS for 5 min, and then incubated in the dark with the secondary antibodies at room temperature (25 °C) for 2 h. The secondary antibodies used were goat anti-mouse IgG H&L (Alexa Fluor® 488) (Abcam, USA, cat#ab150113, 1:500) and goat anti-rabbit IgG H&L (Alexa Fluor® 594) (Abcam, USA, cat#ab150080, 1:500). Another PBS wash was performed, and DAPI (Solarbio, Beijing, China, CAS: 28718–90-3, 1:800) staining was carried out for 10 min. Afterwards, the cells were rinsed with PBS prior to immunostaining. A fluorescence microscope (Olympus, Tokyo, Japan) was employed to capture immunofluorescence images.

### Dot blot analysis

The TaKaRa MiniBEST Universal RNA Extraction Kit (Cat# 9767, Japan) was used to isolate total RNA from the cells as described by the manufacturer. Next, UV spectrophotometry was employed to quantify the RNA.  200 ng RNA samples were loaded onto the GE Healthcare Amersham Hybond-N+ membrane (RPN119B) with a Bio-Rad Bio-Dot Apparatus (# 170–6545) and UV-cross linked to the membrane. Next, 5% dry milk (in 1× PBST) was used to block the membrane for 1–2 h, which was then incubated with a distinct anti-Ψ antibody (MBL, Nagoya, Japan, CODE No. D347–3, 1 μg/mL) overnight at 4 °C. Subsequently, a horseradish peroxidase (HRP)-conjugated secondary antibody was introduced into the blots for 1 h and incubated at room temperature (25 °C). The signal of the dots was visualised using Bio-Rad ChemiDoc XRS+ Imaging System and quantified with Image Lab (Bio-Rad).

### Co-IP, immunopurification, and mass spectrometry

The following antibodies, including Flag (F1804, Sigma), HSP90 (Abcam, USA, cat#ab2928), and PUS7 (Bethyl, USA, A305-147A), were used for the IP assays. DLD1 cells transfected with Flag-PUS7 were immunoprecipitated with anti-Flag magnetic beads and then liquid chromatography-tandem mass spectrometry was employed to process the cells for proteomic data analysis. Co-IP assays were conducted using the Abcam Immunoprecipitation Kit (ab206996) as described by the manufacturer. In brief, the non-denaturing lysis buffer was employed to isolate the total protein lysate. Next, we incubated 500 μg of total proteins with 1 μg of the primary antibody or IgG overnight at 4 °C. Subsequently, the Protein A/G Sepharose® beads were introduced into the mixture and shaken for 1 h at 4 °C. Subsequently, a washing buffer was employed to rinse the beads after which they were diluted in a loading buffer (5×) and denatured by boiling for 10 min. Protein samples were then subjected to WB assay.

### Protein stability

To evaluate protein stability, DLD1 and HCT-8 cells were exposed to 1 μM NMS-E973 for 12 h before the addition of CHX (30 μg/mL). Whole-cell lysates were harvested at specified times. Then, PUS7 protein expression was assayed via WB analysis.

### Databases

Oncomine (https://www.oncomine.org), The Cancer Genome Atlas (TCGA) (https://cancergenome.nih.gov) datasets, and GEPIA were employed to explore the mRNA expression of PUS7 in human cancer samples in contrast to that in non-malignant tissues.

### Quantification and statistical analysis

The SPSS software (V.19.0) and Prism version 9.0 were employed to conduct the data analysis. Fisher’s exact test was used to analyse categorical data. In the intergroup comparisons of quantitative data, the Student’s t-test was applied. Kaplan-Meier survival analysis along with the log-rank test was used to explore the cumulative survival rates. To identify the indicators that independently affected the survival, predetermined variables established to be significant were subjected to multivariate analysis to further analyse them using the Cox proportional hazards model. *P* < .05 signified statistical significance.

## Results

### PUS7 is highly expressed in CRC and is significantly linked to poor prognosis

**Fig. 1:**
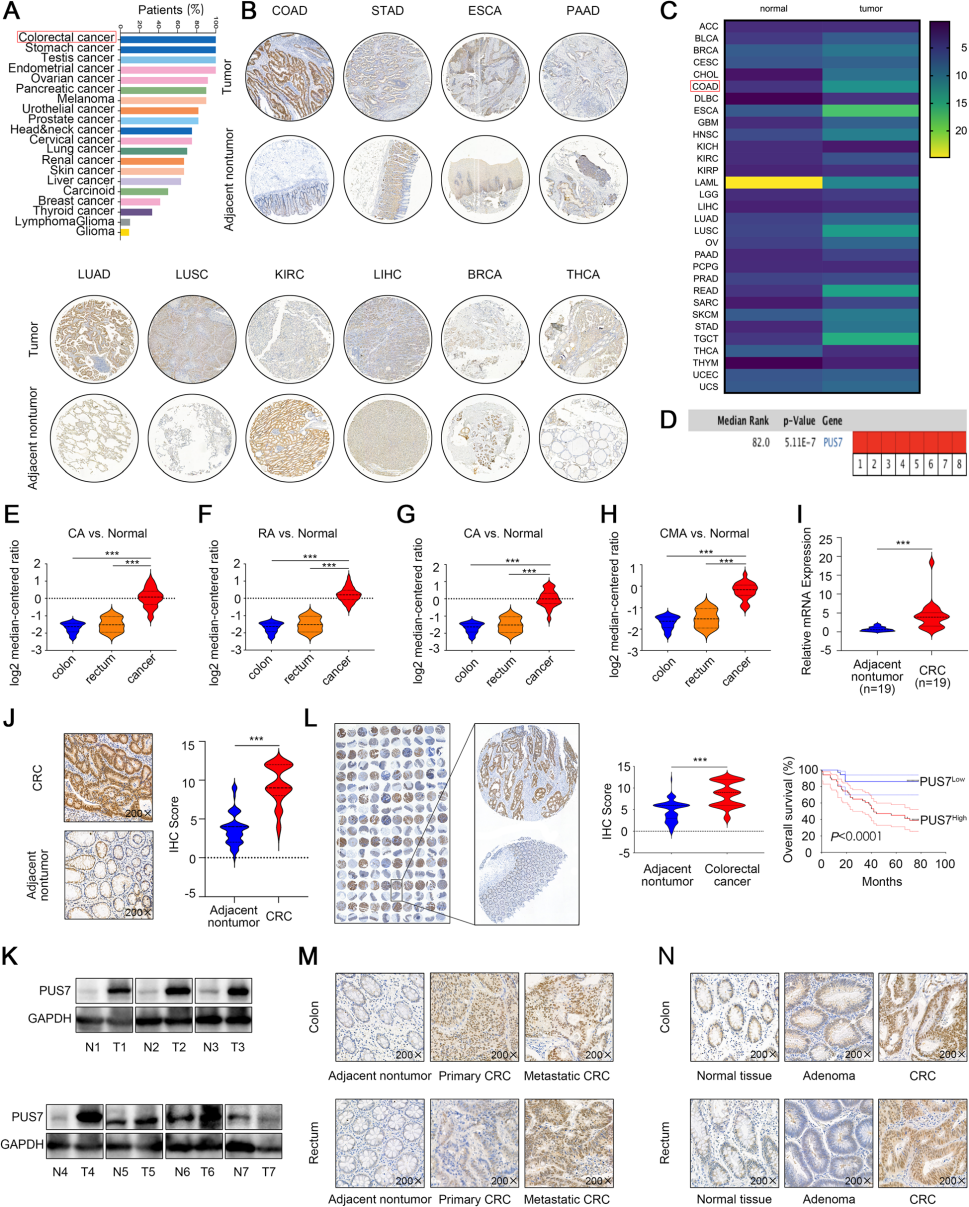
PUS7 is overexpressed in CRC and significantly linked to poor prognosis in CRC patients. **a** Assessment of the relative PUS7 expression in diverse cancer subtypes (HPA024116 dataset). **b** Representative IHC staining of PUS7 expression in LUSC, LUAD, LIHC, THCA, BRCA, KIRC, ESCA, STAD, PAAD, and COAD tissues. **c** *PUS7* expression trend at the mRNA level across different tumour samples and paired non-malignant tissues on the basis of the GEPIA data resource. **d** Meta-analysis of *PUS7* gene expression in the context of five Oncomine data repositories, where coloured squares indicate the median rank for PUS7 (vs. non-malignant tissue) across 8 analyses: Gaspar Colon (1), Kaiser Colon (2), Skrzypczak Colorectal (3), Skrzypczak Colorectal 2 (4), TCGA Colorectal (5–8). The *P* value is given for the median-rank analysis. **e-h** *PUS7* mRNA levels in CRC patients based on TCGA datasets from the Oncomine database. **i** qPCR analysis of *PUS7* expression in 19 in-house collected CRC tissues. **j** Representative IHC staining and plot of IHC-scores of PUS7 expression in 57 in-house collected CRC tissues and neighbouring non-malignant tissues. **k** WB analysis of PUS7 protein expression in 7 CRC tissue pairs; GAPDH was used as the loading standard. N, neighbouring non-malignant tissues; T, tumour tissues. **l** IHC evaluation of CRC and non-malignant neighbouring tissues in a TMA and Kaplan–Meier analysis of the relationship of PUS7 expression with OS in individuals with CRC. **m** Representative IHC staining of PUS7 expression in neighbouring non-malignant tissues, primary CRC tissues, and metastatic CRC tissues. **n** Representative IHC staining of PUS7 expression in neighbouring non-malignant tissues, adenoma tissues, and CRC tissues. All the data are shown as the mean ± s.d. *P < 0.05, **P < 0.01, ***P < 0.001

To gain insights into the expression of PUS7 in different human cancer tissues, we first evaluated the PUS7 protein expression via the analysis of an independent human dataset (HPA024116) retrieved from the HPA database [46]. PUS7 was strongly stained in CRC tissues; in fact, its expression was more commonly observed in CRC tissues than in other cancer subtypes (Fig. 1a). Subsequently, we conducted immunohistochemistry (IHC) staining analysis in different human cancer tissues, including LUSC (Lung squamous cell carcinoma), LUAD (Lung adenocarcinoma), LIHC (Liver hepatocellular carcinoma), THCA (Thyroid carcinoma), BRCA (Breast carcinoma), KIRC (Kidney renal clear cell carcinoma), ESCA ( Esophageal carcinoma), STAD (Stomach adenocarcinoma), PAAD (Pancreatic adenocarcinoma), and COAD (Colon adenocarcinoma). Specifically, we observed strong immunoreactivity in COAD, moderate immunoreactivity in STAD, ESCA, LUAD, LUSC, LIHC, and weak immunoreactivity in PAAD, KIRC, BRCA, and THCA (Fig. 1b).

We then explored the median expression of PUS7 mRNA across all tumour samples and matched non-malignant tissues using the GEPIA [47] website. The heat map in Fig. 1c shows remarkable differences in PUS7 expression in a considerable number of tumour tissues in contrast to those in neighbouring non-neoplastic controls. Further, data retrieved from the Oncomine data repository [48] showed that the median rank of *PUS7* among upregulated genes in CRC was 82 on the basis of a meta-analysis performed across five cohorts, including eight analysis using the Oncomine algorithms (699 samples, *P* = 5.11E-7, Fig. 1d). Importantly, the statistical data demonstrated that PUS7 mRNA was significantly upregulated in CRC tissues in contrast to that in neighbouring non-neoplastic controls (Fig. 1e-h). Consistently, quantitative PCR (qPCR) analysis of 19 CRC specimen pairs (primary tumour and corresponding normal tissues) acquired from the Xijing Hospital of Digestive Diseases revealed that the relative transcription levels of PUS7 were dramatically upregulated in CRC tissues in contrast to those in non-malignant controls (Fig. 1i).

To further examine the expression of PUS7 at the protein level in CRC tissues, IHC analysis was performed in a cohort of 57 CRC specimen pairs (primary tumour and corresponding normal tissues) acquired from the Xijing Hospital of Digestive Diseases. We established that PUS7 was significantly overexpressed in primary CRC tissues (Fig. 1j). Western blotting (WB) further verified the upregulation of PUS7 in 7 pairs of CRC tissues, consistent with the data at the mRNA level (Fig. 1k). Next, we performed IHC analysis of a cohort of TMA, including 89 pairs of CRC specimens, despite a considerable number of cases being high-grade CRC. The data showed that the distribution of the positive staining of PUS7 was predominant in the nucleus and cytoplasm of CRC cells. Of note, of the 28 T4 (depth of invasion) CRC cases, 21 (75.0%) displayed PUS7 overexpression, whereas the remaining 7 (25.0%) exhibited a downregulation of PUS7 expression. Figure 1l illustrates the PUS7 IHC staining trends in CRC. Statistical analysis further confirmed that the PUS7 protein levels were significantly higher in CRC tissues versus neighbouring non-malignant tissues. Importantly, Kaplan-Meier analysis results established that elevated PUS7 expression was significantly linked to a shorter overall survival (OS) in this cohort. Therefore, altogether, these results highlight PUS7 as a prospective prognostic predictor of CRC.

Next, to disclose any correlation between PUS7 expression and CRC pathological features, we compared the protein expression of PUS7 in neighbouring non-malignant, primary, and metastatic sites. The IHC data demonstrated that, in contrast to the neighbouring non-malignant tissues or to the primary site, the metastatic site displayed the highest expression of PUS7 (Fig. 1m). Importantly, PUS7 was upregulated in the context of high clinical stage CRC; for instance, the PUS7 protein levels were dramatically higher in CRC tissues compared to those in adenoma tissues and neighbouring non-malignant tissues (Fig. 1n).

Altogether, these data imply that PUS7 is significantly overexpressed in CRC and is linked to the dismal prognosis of CRC patients.

### PUS7 promotes CRC cell metastasis in vitro and in vivo

**Fig. 2:**
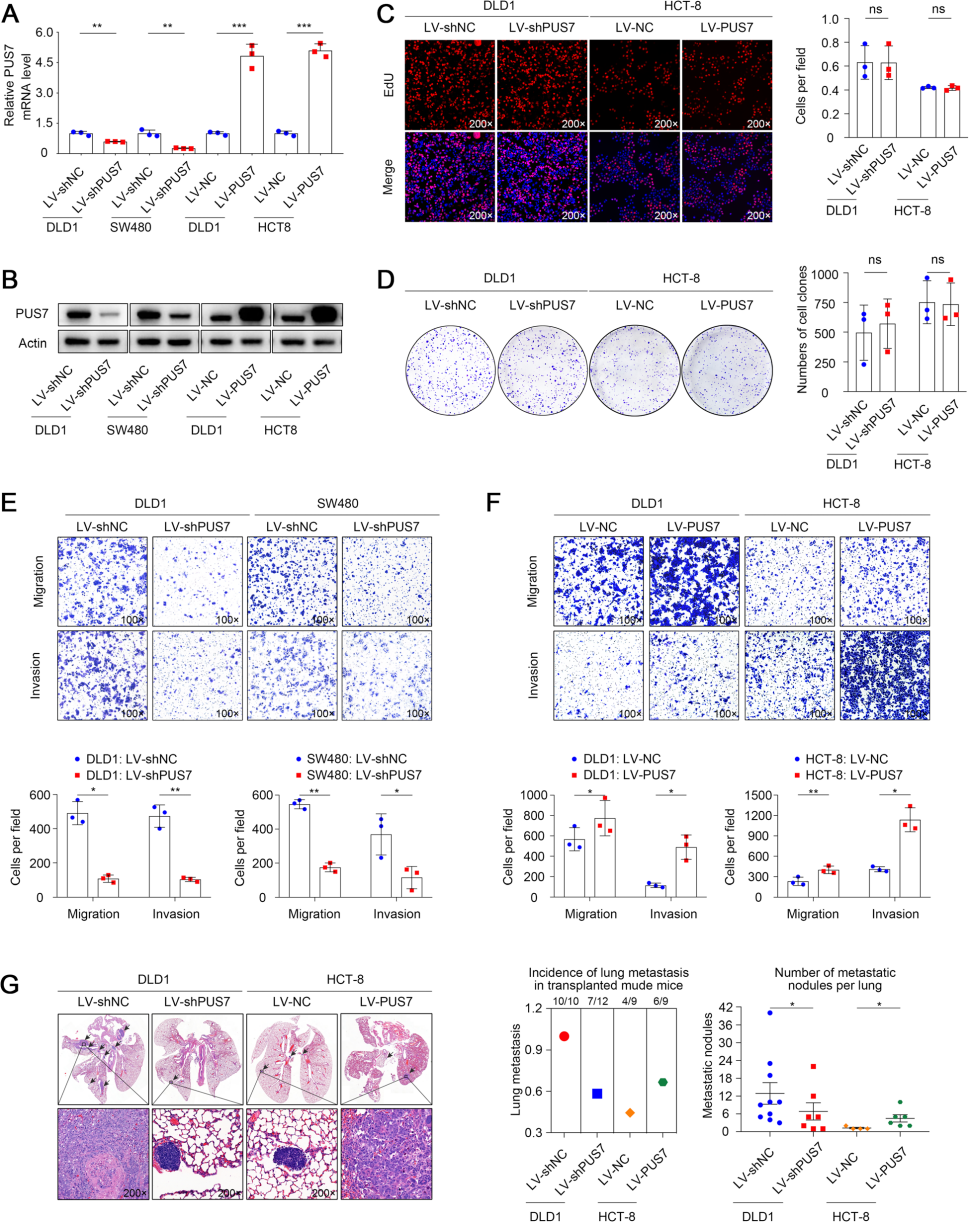
PUS7 regulates the migration and infiltration of colorectal cancer cells in vitro and in vivo*.* **a, b** qPCR **a** and WB **b** analyses of the PUS7 expression in the specified CRC cell lines. **c, d** The influence of PUS7 on CRC cell growth was assessed via EdU proliferation **c** and colony-formation **d** assays in the context of the specified CRC cell lines. **e, f** Transwell assay for the evaluation of cell migration and infiltration using the specified cells. **g** Images illustrating lung tissues stained by H&E from the various groups; the numbers of lung metastatic nodules are indicated (*n* = 15 mice per group for DLD1 cells and *n* = 10 mice per group for HCT-8 cells). All the data are shown as the mean ± s.d. *P < 0.05, **P < 0.01, ***P < 0.001

The contribution of PUS7 to CRC cell function has not been explored to date. Therefore, we elucidated the role of PUS7 in CRC tumorigenesis. To establish the biological role of PUS7 in CRC cells, we downregulated PUS7 expression in DLD1 and SW480 cells and ectopically upregulated PUS7 expression in DLD1 and HCT8 cells. qPCR and WB were performed to evaluate the transfection efficiency (Fig. 2a, b). EdU and colony-formation assays showed that knocking down or overexpressing *PUS7* did not affect the growth of DLD1 and HCT-8 cells (Fig. 2c, d). Transwell assays were also employed to explore the migration capacity of the cells. *PUS7* silencing diminished the migration rates of DLD1 and SW480 cells, whereas the overexpression of *PUS7* exhibited a contrasting trend. Similarly, the Transwell-Matrigel cell invasion assay data confirmed that the infiltration potential of CRC cells was remarkably repressed as a result of *PUS7* silencing, whereas it was remarkably promoted by ectopic *PUS7* expression (Fig. 2e-f). These data imply that PUS7 plays a pivotal role in the migration and invasion of CRC cells.

Next, to evaluate the physiological significance of PUS7 in CRC metastasis in vivo, we injected DLD1-shPUS7 and HCT-8-PUS7 cells into the tail vein of BABL/c nude mice. Eight weeks later, all mice were sacrificed, and their lungs were resected and embedded in paraffin. Histological evaluation showed that the lung metastasis incidence and the number of metastatic nodules in the DLD1-shPUS7 group were dramatically decreased in contrast to those in the control group. On the other hand, PUS7 upregulation significantly increased the incidence of lung metastasis, as well as the proportion of lung metastasis nodules in contrast to that in the HCT-8-control group (Fig. 2g). Altogether, these results suggest that *PUS7* overexpression promotes tumour metastasis in vivo.

### Identification of the PUS7 targets in CRC

**Fig. 3:**
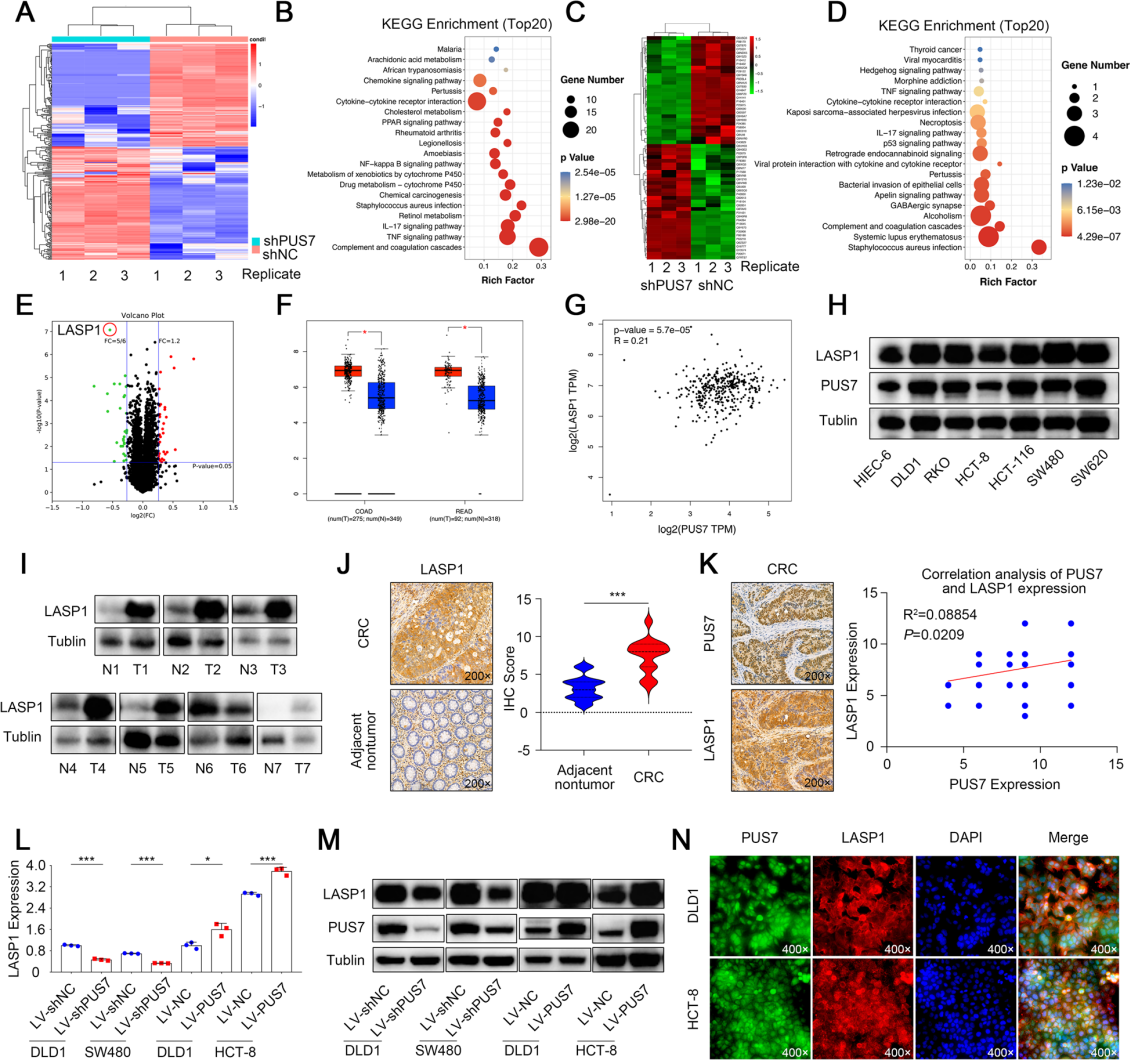
Identification of PUS7 downstream targets in CRC cells. **a** Heatmap of the PUS7 downstream targets assessed by RNA-seq analysis. Red refers to the upregulation of the corresponding genes, while blue refers to the downregulation. **b** Bubble plot of KEGG enrichment terms based on RNA-seq results. The alteration in colour from red to blue illustrates a decrease in the *P*-value, while the size of the circles indicates the number of genes enriched in KEGG terms. **c** Heatmap of the PUS7 downstream targets assessed by proteome profiling analysis. Red refers to the upregulation of the corresponding genes, while green refers to the downregulation. **d** Bubble plot of KEGG enrichment terms based on proteome profiling results. The alteration in colour from red to blue illustrates a decrease in the *P*-value, while the circle size designates the number of genes enriched in KEGG terms. **e** Volcano plot showing the proteins that changed significantly between *PUS7* knockdown DLD1 and control cells. **f** Levels of expression of *LASP1* between tumour and non-malignant tissues of COAD and READ by GEPIA analysis. **g** Correlation analysis of *PUS7* and *LASP1* mRNA expression levels in the GEPIA database. **h** PUS7 and LASP1 expression in the specified cell lines. **i** PUS7 and LASP1 expression in 7 pairs of CRC tumour and non-malignant control tissues. **j** Images illustrating IHC staining for LASP1 in human CRC tissues and neighbouring non-malignant tissues, and corresponding statistical analysis. **k** Images illustrating IHC staining for PUS7 and LASP1 expression in CRC tissues and corresponding correlation analysis. **l, m** qPCR **l** and WB **m** analyses of the LASP1 expression in the specified stable cell lines. **n** Co-localization detection of PUS7 and LASP1 performed by immunofluorescence (IF) analysis

Existing findings showed that PUS7 is a highly intriguing enzyme with the capacity to strongly affect cellular gene expression. To assess changes in global gene expression after knockdown of *PUS7*, RNA-seq analysis was conducted in the context of *PUS7*-silenced DLD1 cells and control cells. *PUS7* depletion resulted in the global alteration of 292 genes: 152 upregulated genes and 140 downregulated genes (Fig. 3a, Supplementary Table S2). KEGG enrichment data illustrated that *PUS7* could alter multiple pathways, including the TNF signalling cascade, IL-17 signalling cascade, chemical carcinogenesis, glycolysis/gluconeogenesis, and complement and coagulation cascades, demonstrating the modulatory roles of PUS7 in CRC tumorigenesis (Fig. 3b).

Given that proteins are the “executioners of life”, and that the abundance of mRNA transcripts does not certainly predict protein abundance differences [49, 50], we further performed proteome profiling analysis using *PUS7* knockdown DLD1 cells and controls.  31 upregulated and 29 downregulated proteins were identified (Fig. 3c, Supplementary Table S3). Consistent with RNA-seq results, KEGG enrichment analysis of differentially expressed proteins showed that PUS7 could also alter the TNF signalling cascade and IL-17 signalling cascade (Fig. 3d). More importantly, PUS7 remarkably modulated multiple metastasis-related genes, including LASP1, S100P, and LCN2 (Fig. 3e). Considering the crucial role of LASP1 in CRC, we decided to focus on the contribution of LASP1 to PUS7-mediated CRC metastasis.

First, data mining from the GEPIA database showed that *LASP1* was not only highly expressed in COAD and READ (Fig. 3f), but also positively correlated with *PUS7* in CRC tissues, as indicated in Fig. 3g. We then assessed the protein expression levels of PUS7 and LASP1 by WB in a panel of CRC cell lines and 7 CRC paired tissues. Importantly, we observed that LASP1 was highly expressed either in CRC cells or CRC tissues (Fig. 3h, i). In addition, IHC analysis of a cohort of 56 pairs of CRC specimens (tumour and matched non-malignant tissues) collected from the Xijing Hospital of Digestive Diseases was performed to verify that LASP1 was overexpressed in CRC tissues and positively linked to PUS7 (Fig. 3j, k).

To confirm the role of PUS7 in the regulation of LASP1 expression in CRC cells, the mRNA and protein expression levels of LASP1 were assayed by qPCR (Fig. 3l) and WB assays (Fig. 3m). The results indicated a positive expression regulation pattern after knockdown or the overexpression of *PUS7* in DLD1, HCT-8, or SW480 cells. As expected, a high *PUS7* expression significantly enhanced the expression of LASP1 protein, while *PUS7* silencing led to a remarkable suppression of LASP1, implying that the LASP1 expression levels are positively modulated by PUS7. Finally, immunofluorescence analysis further confirmed that PUS7 co-localised with LASP1 (Fig. 3n).

Taken together, our results suggest that LASP1 might be regulated by PUS7, which, in turn, promotes CRC metastasis in patients with CRC.

### Increased Ψ level in CRC and PUS7 promote CRC cell metastasis in an RNA pseudouridylation-independent manner

Pseudouridine (Ψ) constitutes a post-transcriptional RNA modification that alters RNA–RNA, and RNA–protein cross-talk, thus influencing gene expression. Experimental data have indicated pivotal roles for pseudouridylation in diverse aspects of gene expression modulation, including spliceosomal small nuclear ribonucleoprotein biogenesis, pre-mRNA splicing efficiency, and translation fidelity [51, 52]. However, the contribution of PUS7 to CRC cell progression in the context of epigenetic RNA modifications was not studied thus far. Therefore, we examined the function of PUS-triggered Ψ during CRC tumorigenesis.

**Fig. 4:**
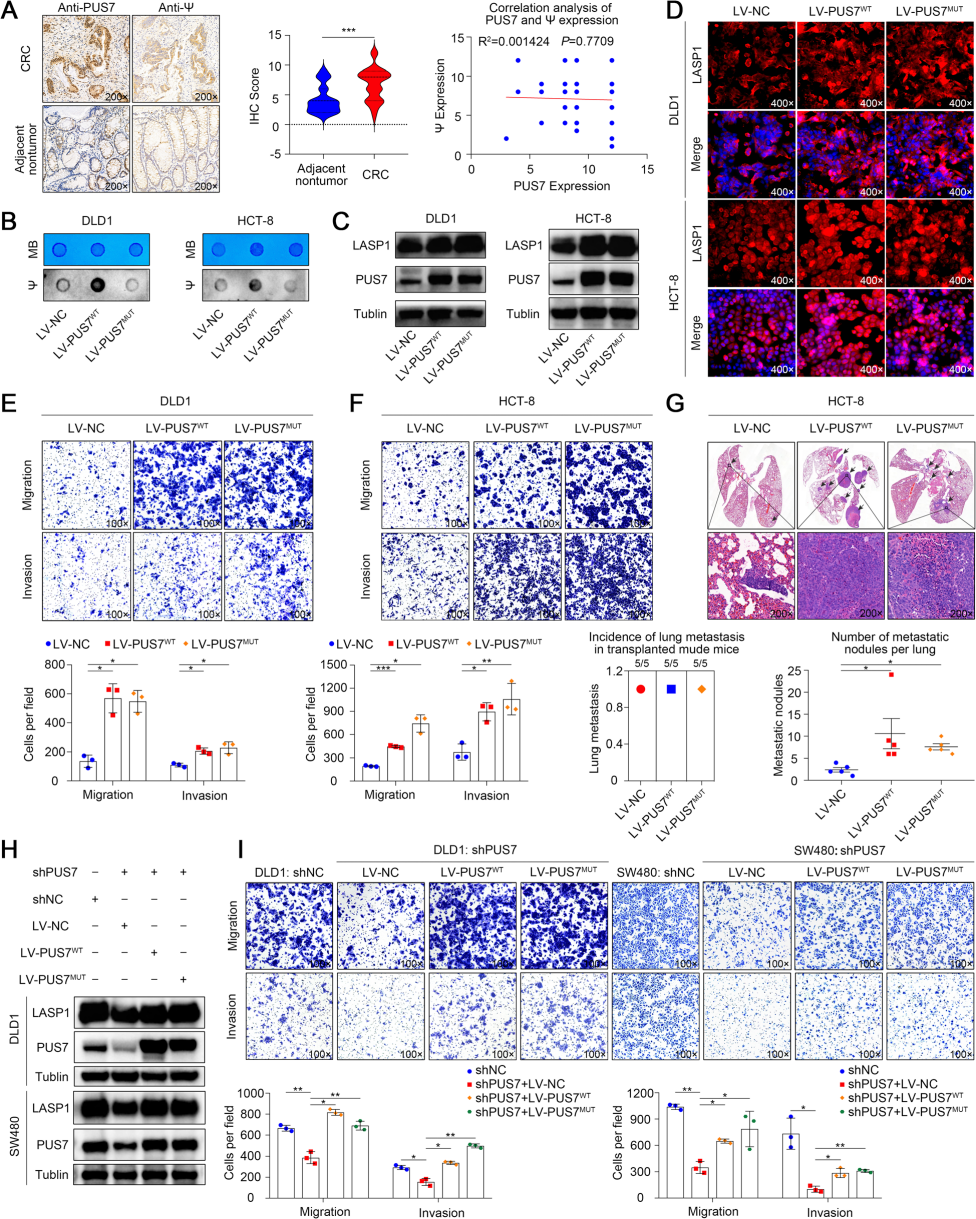
PUS7 promotes CRC metastasis in a catalytically independent manner. **a** Images illustrating IHC staining for Ψ in human CRC paired tissues; the corresponding statistical analysis and correlation analysis between PUS7 and Ψ expression are also shown. **b** Dot plot analysis of the Ψ levels in total RNA extracted from specified stable cell lines. **c** WB analysis of PUS7 and LASP1 expression in the specified stable cell lines. **d** IF analysis of LASP1 expression in the specified stable cell lines. **e, f** Images illustrating Transwell assays in the context of the specified cell lines and the respective statistical analysis. **g** Images illustrating H&E staining of lung tissues from the diverse groups, the incidence of lung metastasis, and statistical analysis (*n* = 5 mice per group). **h** WB analysis of PUS7 and LASP1 expression in the specified stable cell lines. **i** Images of Transwell assay in the context of the specified cell lines and corresponding statistical analysis. All the data are shown as the mean ± s.d. *P < 0.05, **P < 0.01, ***P < 0.001

To study the functional role of Ψ in malignant CRC, we first performed IHC analysis to examine the global Ψ levels in CRC relative to those in non-malignant control samples. As shown in Fig. 4a, we found a strong immunoreactivity localised in the cytoplasm of CRC cells; the calculation of the IHC-score showed a remarkable difference between CRC tissues and surrounding non-malignant tissues (*P* <. 001). However, unexpectedly, the correlation analysis showed no positive relationship between PUS7 and Ψ expression.

Based on these observations, we wondered whether PUS7 could modify the Ψ epi-transcriptome of CRC cells and whether the PUS7’s RNA pseudouridylation activity was necessary. The forced expression of wild-type *PUS7* (LV-PUS7^WT^) and mutant *PUS7* (LV-PUS7^MUT^, with the D294A point-mutation attenuating the PUS7 enzymatic activity) was attained via lentiviral transfection into DLD1 and HCT-8 cells. Dot plot analysis showed the upregulation of the Ψ levels in LV-PUS7^WT^ cells but not in LV-PUS7^MUT^ cells (Fig. 4b). Surprisingly, the protein levels of LASP1 were significantly increased due to the overexpression of *PUS7* (Fig. 4c, d) in both LV-PUS7^WT^ and LV-PUS7^MUT^ cells. However, metastasis and invasion abilities were also significantly increased in both LV-PUS7^WT^ and LV-PUS7^MUT^ cells (Fig. 4e, f), implying that the loss of PUS7-triggered Ψ modifications was not pivotal for the migration and invasion changes in CRC cells. We further injected these stable cells into the tail vein of BALB/c nude mice. The lungs from the sacrificed mice were embedded in paraffin and the metastatic nodules were evaluated. LV-PUS7^WT^ and LV-PUS7^MUT^ animals showed higher metastasis rates than that in the LV-NC group (Fig. 4g).

Furthermore, the decreased expression of LASP1 was rescued to control levels via stable expression of LV-PUS7^WT^ or LV-PUS7^MUT^ in DLD1 and SW480 PUS7-KD cells (Fig. 4h). Consistently, the decreased metastasis ability of PUS7-KD cells was also restored via stable expression of LV-PUS7^WT^ or LV-PUS7^MUT^ (Fig. 4i). These findings verified for the first time that PUS7-mediated Ψ may not govern the metastatic capacity of CRC cells.

### Ectopic expression of LASP1 ameliorates the tumour suppressive effect of PUS7 deficiency in CRC cells

**Fig. 5:**
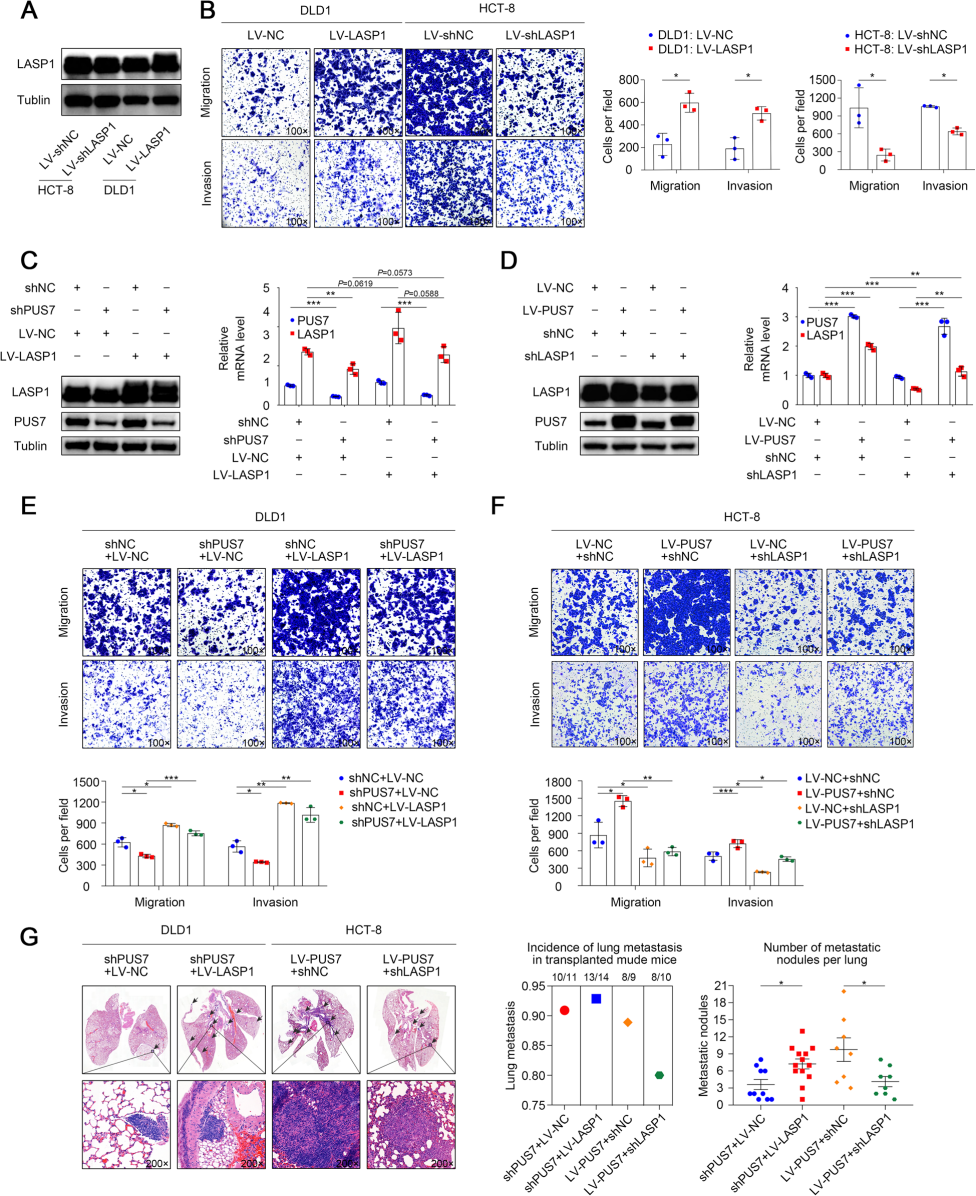
PUS7 promotes CRC metastasis via the upregulation of the expression of LASP1. **a** WB assessment of LASP1 expression in the specified cells. **b** Images illustrating the Transwell assay in the specified cell lines and respective statistical analysis. **c** DLD1 cells were transfected with shPUS7 or *LASP1*. The expression levels of PUS7 and LASP1 were verified by WB analysis and qPCR. **d** HCT-8 cells were transfected with *PUS7* or shLASP1. The expression levels of PUS7 and LASP1 were verified by WB analysis and qPCR. **e, f** Images illustrating the Transwell assay for the specified cell lines and respective statistical analysis. **g** Images illustrating the H&E-stained lung tissues from the diverse groups, the incidence of lung metastasis, and statistical analysis (*n* = 15 mice per group for DLD1 cells and *n* = 10 mice per group for HCT-8 cells). All the data are shown as the mean ± s.d. *P < 0.05, **P < 0.01, ***P < 0.001

Previous studies on different cancer cells, including CRC, have been pivotal for the elucidation of the contribution of LASP1 to tumour metastasis. To verify the key function of LASP1 in CRC metastasis, we conducted a *LASP1* gain-and-loss of function study via transfection of shLASP1 into HCT8 cells and LV-LASP1 into DLD1 cells. The expression of LASP1 in the transfected cells was explored by WB (Fig. 5a). Of note, the Transwell assay data illustrated that *LASP1* downregulation significantly reduced HCT-8 cell migration and invasion, whereas *LASP1* upregulation increased DLD1 cell migration and invasion (Fig. 5b).

To further explore the contribution of LASP1 to PUS7-mediated CRC metastasis, we performed rescue experiments; WB and qPCR confirmed the transfection efficiency (Fig. 5c, d). Transwell assay data illustrated that the migration and infiltration abilities of DLD1 cells repressed by shPUS7 were rescued by the overexpression of *LASP1*, whereas *LASP1* downregulation significantly reduced PUS7-promoted cell migration and infiltration (Fig. 5e, f). Altogether, our data suggest that PUS7 promotes tumour metastasis via LASP1 in CRC cells. Importantly, the data of in vivo metastatic assays showed that *LASP1* overexpression rescued the reduced number of metastatic lung nodules in the DLD1-shPUS7 group, whereas *LASP1* downregulation reduced the number of metastatic lung nodules in the HCT8-PUS7 group (Fig. 5g).

Overall, these data highlight LASP1 as an important functional target of PUS7 and strongly supports the notion that the novel critical signalling axis PUS7/LASP1 is involved in the regulation of CRC metastasis.

### HSP90 is a novel binding partner of PUS7 and increases PUS7 abundance in CRC cells

**Fig. 6:**
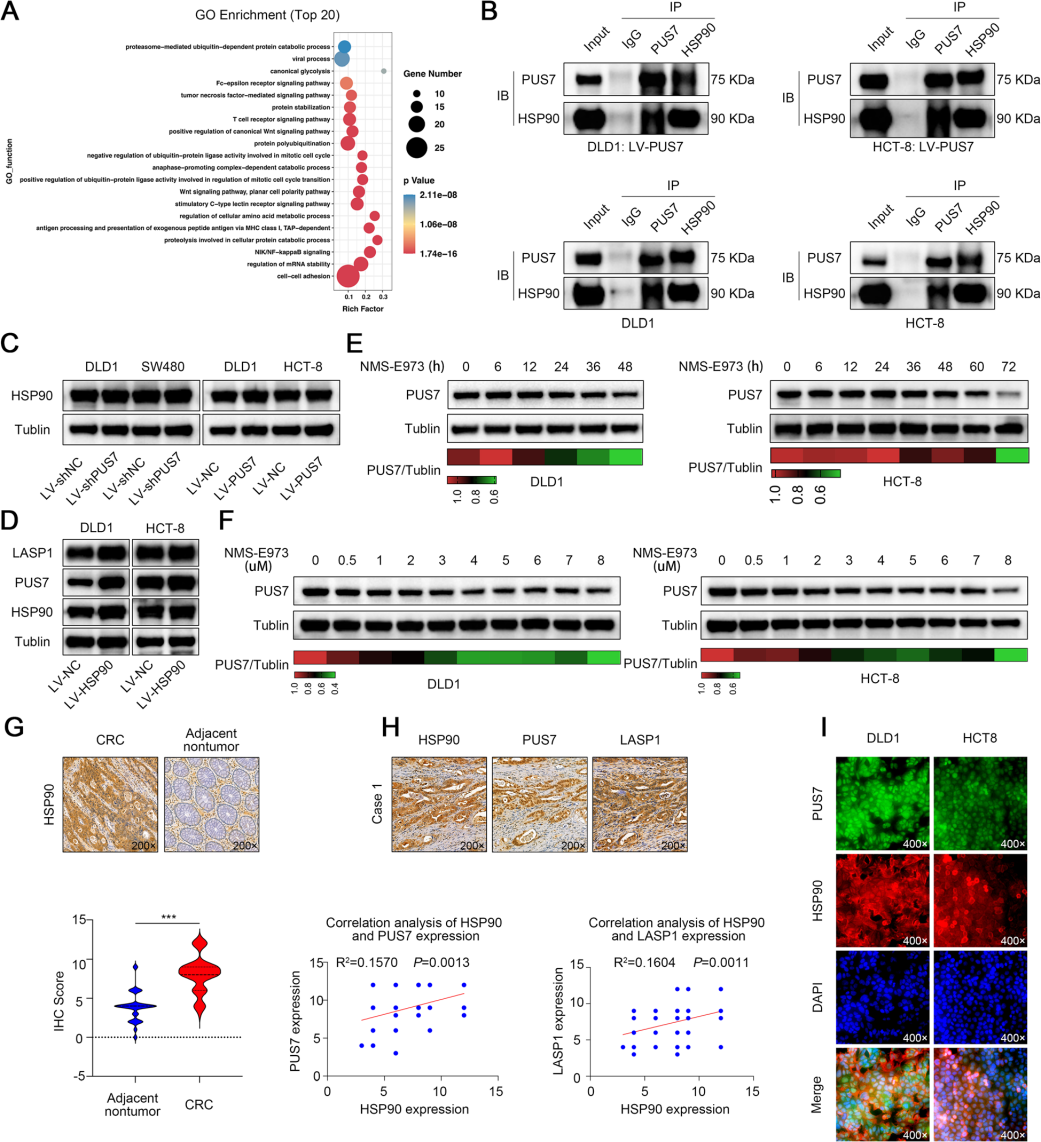
HSP90 binds to PUS7 and increases the PUS7 protein levels in CRC cells. **a** Bubble plot of GO enrichment terms. The alteration in colour from red to blue illustrates a decrease in the *P*-value, while the size of the circles indicates the number of genes enriched in GO terms. **b** DLD1 and HCT-8 were transfected with or without LV-PUS7. Co-IP was performed to determine the relationship between PUS7 and HSP90. **c** WB analysis of HSP90 in the specified cell lines. **d** WB analysis of HSP90, PUS7, and LASP1 in the specified cell lines. **e** DLD1 and HCT-8 cells were treated with NMS-E973 (1 μM) and WB was performed with anti-PUS7 antibodies at the specified time points. **f** DLD1 and HCT-8 cells were exposed to different levels of NMS-E973 as specified and WB was performed with anti-PUS7 antibodies after 24 h of treatment. **g** Selected IHC staining images for HSP90 in CRC matched tissues and relative statistical analysis **h** Selected IHC staining images for HSP90, PUS7, and LASP1 in CRC paired tissues and correlation analysis. **i** Co-localisation of HSP90 and PUS7 performed by IF analysis. All the data are shown as the mean ± s.d. *P < 0.05, **P < 0.01, ***P < 0.001

To elucidate the molecular mechanisms underlying the modulation of PUS7 expression in CRC, we conducted IP-MS (Immunoprecipitation-mass spectrometry) for the PUS7 protein in DLD1-PUS7 cells and identified PUS7-interacting proteins in CRC cells (Supplementary Table S4). Then, we conducted a GO enrichment analysis with the specific interactors of PUS7 (Fig. 6a). In the ranking list of recognised GO functions, we focused on protein stabilization; we reasoned that the factors related to protein stabilization, including CCT3, CCT2, HSP90AA1, HSP90AB1, PARK7, PHB, HSPD1, DSG1, FLNA, CCT8, CALR, PFN1, CCT5, HSPA1B, and HSPA1A, might contribute to PUS7 overexpression in CRC. Previous investigations have indicated HSP90AA1 as a molecular chaperone pivotal for the stability and function of multiple key signalling intermediates (for example, Akt, Raf1, and Erk1/2) [53, 54] important for cell survival, as well as carcinogenesis [55–57]. HSP90-distinct suppressors enhance the degradation of HSP90 client proteins through the ubiquitin/proteasome cascade [53, 54, 58, 59]. We speculated that if PUS7 cross talks directly with HSP90 in CRC cells and is stabilised, then HSP90/PUS7 suppressors could be applied as an effective target for CRC.

To verify the possible interaction between HSP90 and PUS7, suggested by IP-MS, a reciprocal co-immunoprecipitation (Co-IP) assay was conducted on DLD1-PUS7 and HCT-8-PUS7 cell lysates. The data confirmed that PUS7 cross talked with HSP90 directly. Moreover, Co-IP of the endogenous protein confirmed that HSP90 could dock to PUS7 compared to IgG in DLD1 and HCT-8 cells (Fig. 6b).

Next, the influence of PUS7 deficiency on the expression of HSP90 was assessed in CRC cells. The data showed that *PUS7* deficiency caused no remarkable decrease in HSP90 protein; this observation was verified in the *PUS7* overexpression group (Fig. 2c), suggesting that PUS7 may act as a downstream effector of HSP90. Next, we explored whether repressing *HSP90* would influence the PUS7 protein levels in a panel of CRC cells. We found that the overexpression of *HSP90* in DLD1 and HCT-8 cells significantly increased the PUS7 levels (Fig. 6d). On the other hand, the HSP90 inhibitor, NMS-E973, selectively targeted the isoform of HSP90 and caused a time and dose-dependent decrease in the PUS7 protein levels in DLD1 cells and HCT-8 cells (Fig. 6e, f).

To further confirm the positive correlation of HSP90 with the abundance of PUS7 in CRC, IHC staining was conducted in CRC paired tissues collected in-house. The findings illustrated that the HSP90 protein levels were significantly increased in contrast to those in non-malignant tissues (Fig. 6g). Besides, CRC tissues with high HSP90 levels exhibited elevated protein expression of PUS7 or LASP1 in contrast to those with low HSP90 levels. Importantly, Spearman correlation analysis demonstrated a positive relationship between HSP90, and PUS7 or LASP1 IHC scores (Fig. 6h). Lastly, IF analysis demonstrated that HSP90 co-localised with PUS7 in the cytoplasm and nucleus (Fig. 6i). Collectively, these data suggest that HSP90 binds to PUS7 and increases the PUS7 expression in CRC.

### HSP90 enhances the stability and inhibits the proteasome degradation of PUS7 in CRC cells

HSP90 serves as a molecular chaperone and has been implicated in the stabilization of a number of transcription factors, protein kinases, and oncogenic proteins in tumour signalling cascades [60, 61]. Therefore, we speculated that HSP90 participates in the stability of its novel target protein, PUS7.

**Fig. 7:**
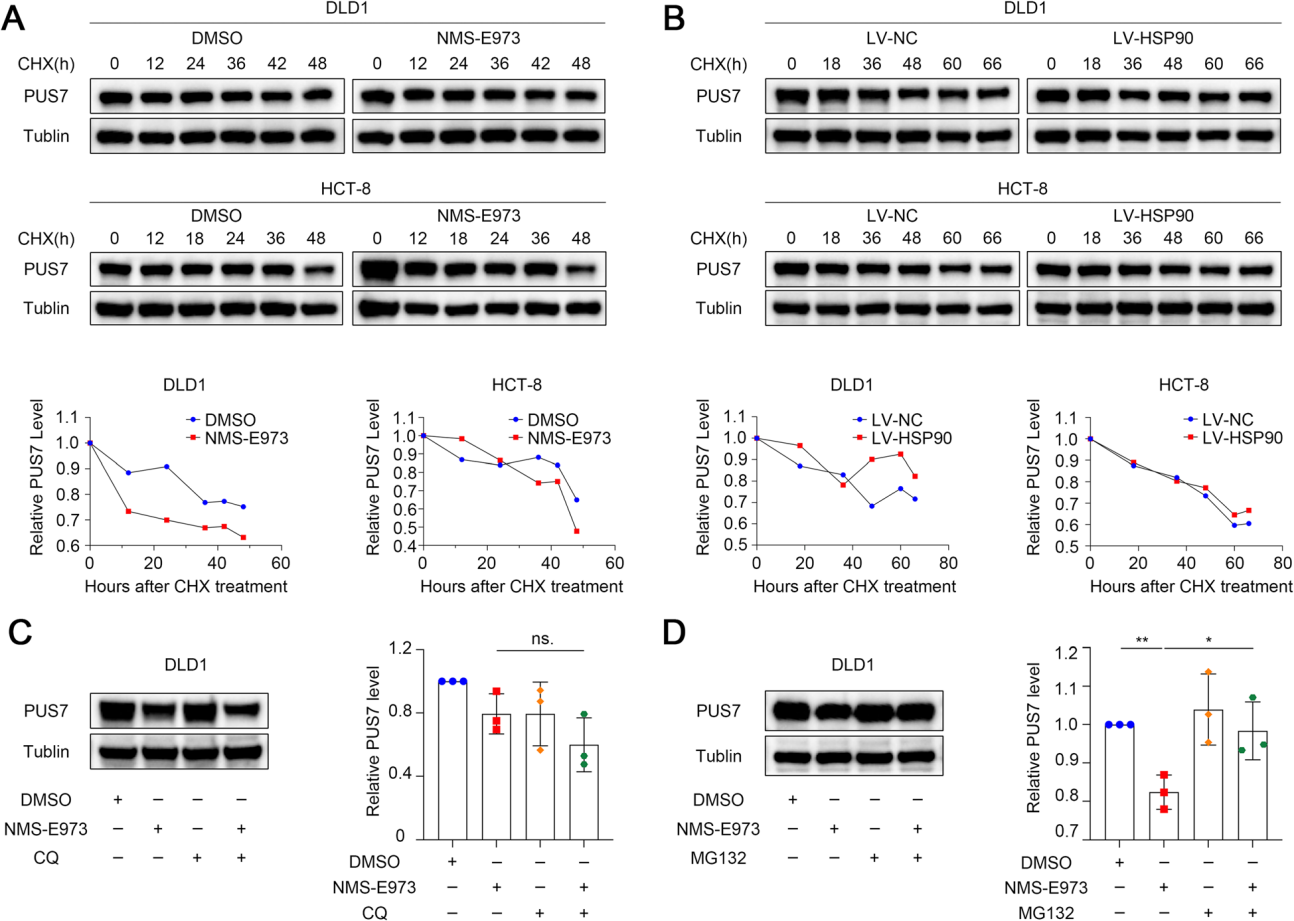
HSP90 regulates the protein stability of PUS7 in CRC cells. **a** CHX-chase assay showing the degradation of PUS7 in DLD1 and HCT-8 cells treated with NMS-E973 (1 μM) or control DMSO. **b** CHX-chase assay showing the degradation of PUS7 in DLD1 and HCT-8 cells transfected with LV-HSP90 or control LV-NC lentivirus. **c, d** CQ (20 μM) was used to inhibit lysosomal degradation **c** and MG-132 (100 nM) was employed to repress proteasomal degradation **d** in DLD1 cells. MG-132 treatment reversed the downregulation of the PUS7 protein levels triggered by *HSP90* suppression. All the data are shown as the mean ± s.d. *P < 0.05, **P < 0.01, ***P < 0.001

To determine whether HSP90 increases the PUS7 levels in CRC cells via the modulating of protein stability, the cycloheximide (CHX, a protein synthesis inhibitor) chase assay was employed. As indicated in Fig. 7a, NMS-E973 pre-incubation markedly destabilised and reduced the half-life of PUS7. Importantly, the PUS7 protein levels were remarkably decreased following treatment with CHX in control DLD1 and HCT8 cells, while the overexpression of *HSP90* partly recovered the CHX-triggered decrease in PUS7 (Fig. 7b).

Usually, HSP90 client proteins are subjected to proteasomal degradation in response to HSP90 inhibitors. To verify this phenomenon, we co-incubated the selective lysosomal inhibitor chloroquine (CQ) or the proteasome inhibitor (MG132) with NMS-E973 in DLD1 and HCT-8 cells. The results showed that CQ had no effect on PUS7 (Fig. 7c), while MG132 significantly restored the decrease in the PUS7 protein levels induced by *HSP90* repression (*P* < 0.05, Fig. 7d). This suggests that HSP90 may elevate the stability of PUS7 via the repression of the proteasome-triggered degradation of PUS7 in CRC cells.

### HSP90 promotes the migration and infiltration of CRC cells in a PUS7/LASP1 axis-dependent manner in vitro and in vivo

**Fig. 8:**
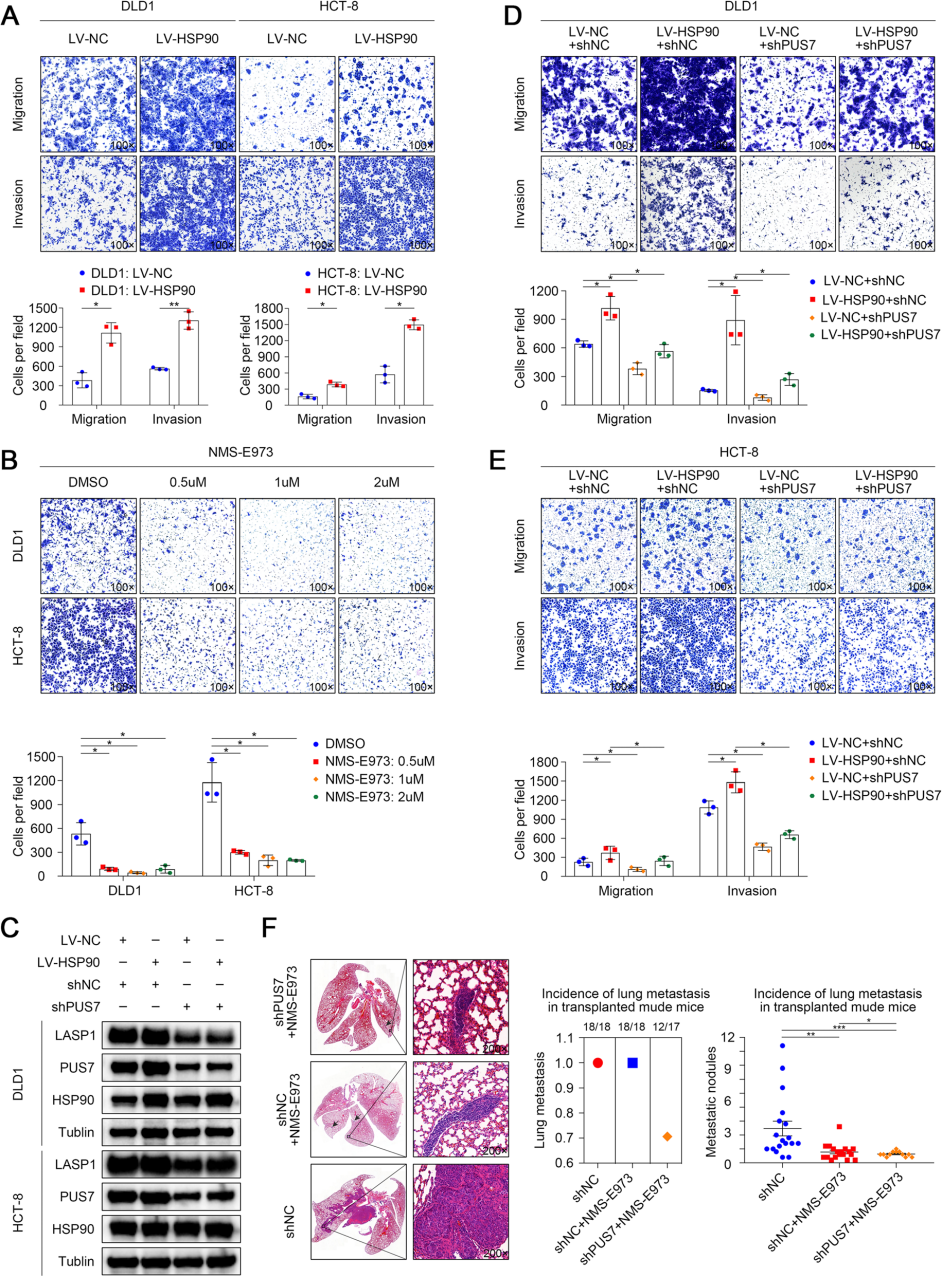
PUS7 mediates the biological role of HSP90 in CRC cells. **a** Images of the Transwell assay for the specified cell lines and corresponding statistical analysis. **b** Images of the Transwell assay for the specified cell lines treated with DMSO or NMS-E973 and corresponding statistical analysis. **c** WB analysis of HSP90, PUS7, and LASP1 expression in the specified stable cell lines. **d, e** Images of the Transwell assay for the specified cell lines and corresponding statistical analysis. **f** Images illustrating H&E-stained lung tissues from the diverse groups, the incidence of lung metastasis, and statistical analysis (*n* = 20 mice per group). All the data are shown as the mean ± s.d. *P < 0.05, **P < 0.01, ***P < 0.001

To assess the effect of HSP90 on CRC cell migration and infiltration, we overexpressed *HSP90* in DLD1 and HCT8 cells through lentiviral transduction. The Transwell assay results showed that *HSP90* overexpression resulted in a higher migration rate in DLD1 and HCT-8 cells. Similarly, the Transwell-Matrigel invasion assay data verified that the infiltration ability of CRC cells was significantly increased in response to *HSP90* overexpression. To investigate the antitumor activity of NMS-E973 against CRC cells in vitro, we further treated DLD1 and HCT-8 cells with NMS-E973 at various concentrations and then measured their infiltration ability. As shown in Fig. 8b, NMS-E973 treatment evidently diminished the invasion ability of DLD1 and HCT-8 cells.

HSP90 has been reported to influence the activation of numerous key signalling intermediates, such as Akt, Erk1/2, and Lck [53, 54, 59, 62]. To determine whether PUS7 mediated the biological role of HSP90 in CRC cells, we conducted rescue experiments and the results showed that depletion of *PUS7* rescued the *HSP90* OE-induced increase in LASP1 protein levels (Fig. 8c) and cell metastasis (Fig. 8d, e) in both DLD1 and HCT-8 cells.

Next, to assess the effect of PUS7 inhibition combined with NMS-E973 on tumour metastasis in vivo, we injected DLD1-shPUS7 and DLD1-shNC cells into the tail vein of BABL/c nude mice. Mice were then administered 10 mg/kg NMS-E973 intraperitoneally every 2 days, starting 2 weeks after CRC cell transplantation. Eight weeks later, all mice were sacrificed, and their lungs were resected, embedded in paraffin, sectioned, and subjected to histological evaluation to detect lung metastasis. The PUS7-KD DLD1 tumours treated with NMS-E973 showed the lowest number of metastatic nodules in contrast to that in the control group. DLD1-shPUS7 cells were more sensitive to NMS-E973 therapy than the control group (Fig. 8f). This implies that HSP90 inhibitors are promising candidates for combination therapy with PUS7-targeting agents in the context of CRC. Further, these data implied that PUS7 is important for the NMS-E973-induced repression of CRC metastasis in vivo and in vitro and that HSP90 inhibitors combined with PUS7 suppression exhibit an activity superior to that of monotherapy targeting HSP90.

### Co-expression of HSP90, PUS7, and LASP1 predicts poor prognosis in CRC patients

**Fig. 9:**
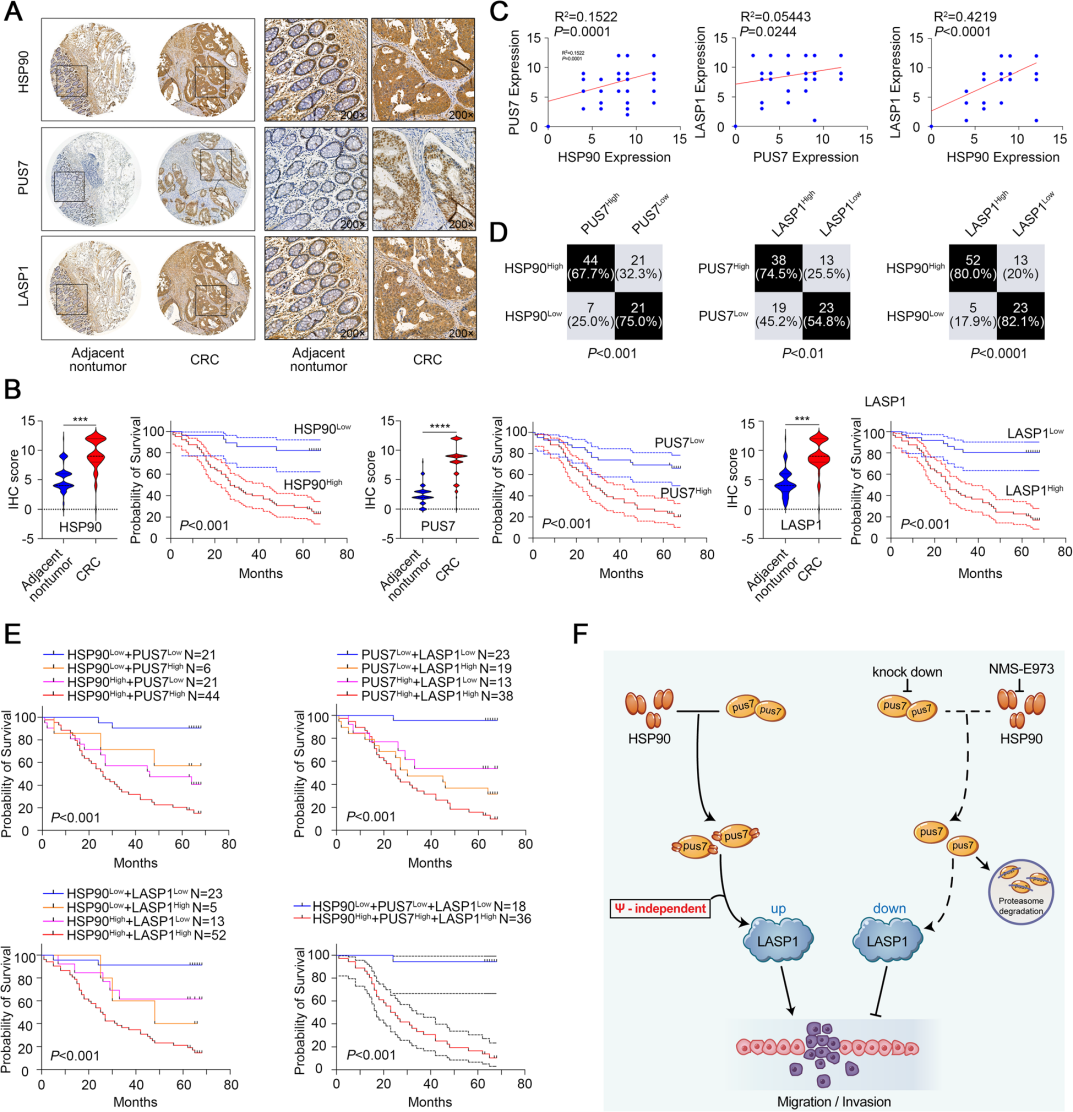
HSP90 expression is significantly linked to PUS7 and LASP1 expression in human CRC. **a** Selected IHC staining images for the expression of HSP90, PUS7, and LASP1 in CRC paired tissues and the respective correlation analysis. **b** IHC score for HSP90, PUS7, and LASP1 in CRC paired tissues and corresponding Kaplan-Meier plotter. **c** Correlation analysis among HSP90, PUS7, and LASP1 in a cohort. **d** Statistical analysis of CRC tissues under diverse staining conditions in a cohort. **e** Kaplan-Meier analysis based on the HSP90, PUS7, and LASP1 expression. **f** A schematic model of the role of the HSP90-PUS7-LASP1 axis in CRC metastasis. HSP90 is a novel binding partner of PUS7 and increases PUS7 abundance via the inhibition of the proteasome-mediated degradation of PUS7 in CRC cells. Upregulated PUS7 markedly enhances CRC cell migration and invasion abilities via the regulation of LASP1 in a catalytically independent manner. Of note, PUS7 inhibition in combination with HSP90 inhibitors (NMS-E973) displays superior anti-metastatic activity in CRC

From the analysis above, IHC staining was employed to confirm the clinical significance of HSP90, PUS7, and LASP1 in a human CRC cohort (*N* = 87). Representative images of the IHC staining are presented in Fig. 9a. We found that the HSP90, PUS7, and LASP1 protein levels were significantly elevated in contrast to those in corresponding normal tissues (Fig. 9b). Next, we clustered the patients into high and low expression groups based on the median value of the IHC-score of HSP90, PUS7, and LASP1. The Kaplan-Meier analysis data showed that a higher expression of HSP90, PUS7, and LASP1 correlated with a lower OS (Fig. 9b). Further, the correlation analysis indicated that the expression of HSP90, PUS7, and LASP1 was significantly correlated with each other (Fig. 9c). Specifically, CRC tissues with high HSP90 levels showed elevated PUS7 and LASP1 protein levels in contrast to those in tissues with low HSP90 levels. Similarly, the specimens with higher PUS7 expression exhibited strong staining for LASP1. Additionally, samples with low HSP90 expression showed low PUS7 and LASP1 levels. Of note, the data were statistically significant (Fig. 9d, *P* < 0.01). Patients were then clustered into four subgroups based on HSP90/PUS7 expression, PUS7/LASP1 expression, or HSP90/LASP1 expression. Kaplan-Meier analysis data showed that patients with either HSP90/PUS7, PUS7/LASP1, or HSP90/LASP1 co-expression had the shortest OS times among all the subgroups. Finally, we clustered the patients into two subgroups based on HSP90/PUS7/LASP1 expression. Kaplan-Meier analysis confirmed that the high expression of HSP90/PUS7/LASP1 predicted the poorest OS (Fig. 9e), suggesting that the HSP90/PUS7/LASP1 regulatory axis is critical to the prognosis of CRC patients.

Additionally, to assess the correlations between HSP90/PUS7/LASP1 expression and clinicopathological features, we employed IHC score to categorize the patients into low and high expression groups based on the median value. The associations between HSP90/PUS7/LASP1 expression and the clinicopathological parameters of CRC are presented in Table 1. High HSP90 positive staining was considerably linked to the pathological stage, T classification, N classification, and clinical stage. Similarly, high PUS7 positive staining was significantly linked to T classification, and high LASP1 positive staining was significantly linked to tumour size, T classification, and clinical stage. Altogether, these data demonstrated the significance of the HSP90/PUS7/LASP1 cascade in CRC development.

**Table 1:** Correlation between the HSP90/PUS7/LASP1 expression and clinicopathological characteristics in human CRC tissues

Variables	Cases (No.) (***n*** = 93)	HSP90		***P***	PUS7		***P***	LASP1		***P***
		Low expression	High expression		Low expression	High expression		Low expression	High expression	
**Age (years)**				0.562			0.780			0.210
< 60	34	9	25		16	18		16	18	
≥ 60	59	19	40		26	33		20	39	
**Gender**				0.128			0.533			0.218
Male	52	19	33		22	30		23	29	
Female	41	9	32		20	21		13	28	
**Pathological stage**				**0.044**			0.157			0.836
Stage I	2	2	0		2	0		1	1	
Stage II	73	23	50		30	43		29	44	
Stage III	18	3	15		10	8		6	12	
**Tumor size**				0.528			0.499			**0.042**
< 5 cm	48	16	32		23	25		23	25	
≥ 5 cm	44	12	32		18	26		12	32	
**T stage**				**0.034**			**0.030**			**0.005**
T2	5	3	2		4	1		5	0	
T3	64	21	43		30	34		24	40	
T4	16	1	15		3	13		3	13	
**N stage**				**0.032**			0.249			0.093
N0	51	21	30		22	29		24	27	
N1	28	7	21		15	13		7	21	
N2	9	0	9		2	7		2	7	
**Metastasis**				0.18			0.408			0.104
0(no)	89	28	61		41	48		36	53	
1(yes)	4	0	4		1	3		0	4	
**Clinical stage**				**0.034**			0.547			**0.008**
Stage I	4	3	1		3	1		4	0	
Stage II	41	16	25		18	23		18	23	
Stage III	35	7	28		16	19		9	26	
Stage IV	4	0	4		1	3		0	4	

Univariate and multivariate analysis were further employed to investigate the prognostic value of HSP90/PUS7/LASP1 expression in CRC (Table 2). The univariate Cox regression results illustrated that pathological stage, N classification, clinical stage, HSP90 expression, PUS7 expression, and LASP1 expression were positive prognostic factors (*P* < .001). The multivariate Cox regression data also suggested that the pathological stage, PUS7, and LASP1 were independent prognostic risk factors (*P* < .05) (Fig. 9m).

**Table 2:** Univariate and multivariate analysis of the factors associated with overall survival in CRC patients

Variables	OS		
	***p***	Hazard Ratio	95% confidence interval
**Univariate analysis**			
Age (< 60 vs. ≥ 60)	0.238	1.422	0.792–2.554
Gender (Male vs. Female)	0.374	1.274	0.746–2.177
Pathological stage (I-II vs. III)	< 0.001	2.964	1.671–5.259
tumor size (< 5 cm vs. ≥ 5 cm)	0.086	1.605	0.935–2.755
T classification (T2-T3 vs. T4)	0.207	1.539	0.788–3.007
N classification (N0 vs. N1-N2)	< 0.001	2.912	1.668–5.082
M classification (absent vs present)	0.099	2.380	0.850–6.667
Clinical stage (I-II vs III-IV)	< 0.001	3.060	1.720–5.442
HSP90	< 0.001	6.869	2.725–17.317
PUS7	< 0.001	3.470	1.876–6.419
LASP1	< 0.001	6.974	3.133–15.527
**Multivariate analysis**			
Pathological stage (I-II vs. III)	< 0.001	3.867	1.870–7.996
N classification (N0 vs. N1-N2)	0.063	0.706	0.162–3.080
Clinical stage (I-II vs III-IV)	0.734	3.117	0.682–14.243
HSP90 (high vs. low)	0.336	1.959	0.579–6.628
PUS7 (high vs. low)	0.003	3.304	1.535–7.108
LASP1 (high vs. low)	0.019	2.923	1.136–7.515

Overall, the above results show that HSP90, PUS7, and LASP1 are upregulated in CRC and positively correlate with each other. Moreover, HSP90/PUS7/LASP1 and the combination of these three proteins estimate the prognosis of CRC patients.

## Discussion

As one of the most profound features of CRC, distant metastasis constitutes a complex process influenced by various oncogenic pathways and is responsible for more than 90% of CRC-related deaths [3]. However, the potential involvement of PUS7 in the context of CRC metastasis is poorly defined. Herein, we report, for the first time, that PUS7 can promote the metastasis of CRC cells through a combination of bioinformatics analysis, multi-omics analysis, and experimental validation. In brief, the upregulation of PUS7 markedly enhanced CRC cell migration and invasion abilities in cultured cells or in mouse models via the regulation of LASP1 in a catalytically independent manner. Crucially, we identified that PUS7 is a protein client of HSP90, which increased the PUS7 protein stability and elevated PUS7 expression in CRC. Besides, the combination of NMS-E973 and PUS7 inhibition showed greater anti-metastatic activity in the context of CRC cells. Importantly, clinical data illustrated that CRC tissues exhibited higher HSP90, PUS7, and LASP1 levels than did the surrounding normal tissues, leading to a poor prognosis in CRC patients. Moreover, PUS7 is prominently highly expressed in T4 CRC tissues as 75.0% in cohort I and 81.25% in cohort II. The metastatic site also displayed the highest expression of PUS7 in contrast to the neighboring non-malignant or primary site. Consistently, PUS7 expression was higher in HCT-116 and SW620 cells (with high metastatic ability) than in HCT-8 and SW480 cells (with low metastatic ability), indicating that PUS7 might be exclusively important for Grade IV CRC. Altogether, these results reveal a new critical role of the HSP90/PUS7/LASP1 axis in the promotion of CRC cell metastasis and may serve as a valuable prognostic biomarker to distinguish CRC patients with advanced-stage disease and/or poor survival.

Previous studies have demonstrated a role for PUS7-mediated pseudouridylation in translation and tumorigenesis [21]. However, the role of PUS7 in CRC development remains poorly understood. In this study, we found that PUS7 enhanced the metastasis of CRC via the modulation of LASP1 in an RNA pseudouridylation-independent manner. Of note, PUS7 affects CRC cell metastasis, but not proliferation, as per our functional assay. Similarly, a previous study found that PUS7 depletion did not affect cell proliferation but led to an increase in cell size in hESCs [21]. PUS10 also affects cell growth in multiple human cell lines [13]. These results not only stress the importance of PUSs in cellular processes but also provide suggest tissue-specificity in the biological function of PUSs. As there is little information available on the function of individual pseudouridylation enzymes, future studies are needed to determine the role of PUSs in the context of different cell phenotypes.

Previous studies have highlighted PUS7 as a Ψ “writer” that catalyses pseudouridine formation [63], impacting various aspects of gene expression modulation, including translation fidelity [52]. For example, the PUS7-triggered Ψ modifications of tRNA-derived small fragments could regulate protein biosynthesis in embryonic stem cells [21]. However, our functional study and rescue experiments showed that PUS7 promotes CRC metastasis and regulates the expression of LASP1 in a catalytically-independent manner, meaning that the Ψ marks seem to be dispensable for the dysregulation of LASP1 in CRC. Interestingly, the nuclear PUS10 promotes miRNA processing independently of its catalytic activity as well [13]. Moreover, METTL3, an m6A “writer”, promotes translation independently of its methyltransferase activity [64]. Thus, our results imply that PUS7 may act as an RNA-binding protein, highlighting it as a new example of the catalytically independent roles of PUS enzymes in CRC development. In support of this hypothesis, a recent study identified a new putative binding motif “GTT [CTA][GA]A” on RNAs bound to PUS7 in hESCs [21], suggesting that PUS7 may have a complex RNA-binding capacity. Since both Ψ modifications and the catalytically independent roles of PUSs have not been well studied, further studies are needed to understand whether PUS7 contributes to other malignancies in a Ψ-dependent or -independent manner.

LASP1 was defined as a complex nuclear transcriptional regulator and a metastasis-related protein in CRC and other solid tumours [32, 65–67]. Of note, previous research only identified a list of miRNAs contributing to the regulation of LASP1 in CRC [23]. Herein, we demonstrated that LASP1 functions as a downstream target of PUS7 and is functionally responsible for PUS7-mediated CRC metastasis, providing a new regulatory layer. However, the gene regulatory networks of PUS7 are diverse and complex. For example, our RNA-seq and proteome profiling data illustrated that PUS7 could alter the TNF signalling cascade and IL-17 signalling cascade. Whether HSP90 affects TNF/IL-17 signalling through PUS7 and the relationship between LASP1 and TNF/IL-17 signalling in CRC remain to be verified. Moreover, whether PUS7 regulates the LASP1 mRNA expression directly or works on other types of RNA in a catalytically independent manner, affecting, in turn, the LASP1 expression, is far from being understood. Therefore, we cannot rule out the involvement of other genes in the PUS7-mediated biological functions.

Despite the observation that PUS7 is highly expressed in CRC tissues, the mechanism behind the upregulation of PUS7 was, thus far, unknown. In this study, we identified HSP90 as a novel binding partner of PUS7, essential for its upregulation in CRC. Of note, PUS7 was reported to be regulated by heat shock and some other stress conditions [16, 37]. HSP90 is a molecular chaperone upregulated by heat shock and modulates the stability of multiple oncogenic proteins [68–71]. Furthermore, the PUS7 mRNA levels decreased under rapamycin treatment [72], which targets the mTOR pathway involved in metabolism and tumorigenesis [73]. Importantly, HSP90 is closely associated with the mTOR pathway [74]. Thus, we hypothesized that HSP90 may also regulate PUS7 transcripts via the mTOR pathway, something we intend to validate in our next experiments. Importantly, our CHX assay confirmed that HSP90 could elevate the stability of PUS7 via the repression of the proteasome-triggered degradation of PUS7 in CRC cells. Of note, PUS7 was reported to be modified by either ubiquitination or succinylation [38–40]. Thus, we hypothesise that PUS7 may be ubiquitinated in CRC cells. We intend, in a follow-up study, to investigate in-depth the mechanisms of the regulation of the expression of PUS7.

With the aim of discovering a pharmacological approach against the HSP90/PUS7/LASP1 axis, we focused on one of the HSP90 selective inhibitors, NMS-E973, due to the lack of available PUS7 inhibitors. Notably, HSP90 is considered a prospective therapeutic target in the treatment of cancers driven by oncoproteins, such as HER2, CDK4, BRAF, EGFR, MET, CRAF, AKT, EML4-ALK, and BCR-ABL [75, 76]. However, many clinical trials have been terminated or postponed due to the observation of moderate effects or due to toxicity based on low specificity (clinicaltrials.gov). Previously, it was suggested that the combination of HSP90 inhibitors with targeted agents may strengthen the effect of anti-neoplastic treatments [77–79]. Importantly, our results aligned with these claims; NMS-E973 exhibited remarkable antitumor efficacy against CRC in vitro, and the combination of NMS-E973 with the downregulation of PUS7 significantly reduced CRC metastasis in contrast to NMS-E973 monotherapy in vivo. Therefore, HSP90 suppressors are promising combination partners in the context of PUS7 blockade agents for the treatment of PUS7-overexpressing tumours.

Some limitations should be considered in this study. First, the number of human CRC cases in our cohorts, especially the proportion of metastatic patients, were relatively insufficient, which makes PUS7 seem insignificant for the distinction of higher-clinical stage and lower-clinical stage tumours. Second, we did not construct orthotopic CRC mouse model to specify the metastatic potential of PUS7-overexpressing cells. Third, the underlying mechanisms between PUS7 and LASP1 still remain unclear. Thus, a prospective study with larger CRC cohort, an orthotopic CRC mouse model and in-depth molecule mechanisms about HSP90/PUS7/LASP1 in CRC should be addressed and clarified in the future.

## Conclusion

In conclusion, we report, for the first time, that PUS7 can promote CRC metastasis via the regulation of LASP1, independently of its Ψ enzyme activity. Further, HSP90 was revealed as a novel binding partner of PUS7, promotes the protein abundance of PUS7 in CRC. NMS-E973 showed increased anti-tumour metastasis activity when combined with PUS7 suppression in CRC. Hence, the mechanistic comprehension of the HSP90/PUS7/LASP1 axis in CRC metastasis will open novel research opportunities for the elucidation of the functional consequences of PUS7 in the context of tumour development and for the development of new treatment approaches for CRC.

## Supplementary Information

**Additional file 1: Supplementary Table 1.** Primer sequences used in the study. **Supplementary Table 2.** List of genes differentially expressed in DLD1-PUS7-KD versus DLD1-control cells using RNA-Seq. **Supplementary Table 3.** List of proteins differentially expressed in DLD1-PUS7-KD versus DLD1-control cells using Proteome-profiling. **Supplementary Table 4.** List of proteins potentially interacted with PUS7 and IgG in DLD1-PUS7-OE cells using Immunoprecipitation-mass spectrometry. **Supplementary Table 5.** Description of the patient samples acquired from clinical datasets of Xijing Hospital of Digestive Diseases

## Data Availability

All data generated or analysed during this study are included in this published article and its supplementary information files.

## Abbreviations

PUS7: Pseudouridine synthase 7
OS: overall survival
TCGA: The Cancer Genome Atlas
GEPIA: Gene Expression Profiling Interactive Analysis
CRC: Colorectal cancer
LUSC: Lung squamous cell carcinoma
LUAD: Lung adenocarcinoma
LIHC: Liver hepatocellular carcinoma
THCA: Thyroid carcinoma
BRCA: Breast carcinoma
KIRC: Kidney renal clear cell carcinoma
ESCA: Esophageal carcinoma
STAD: Stomach adenocarcinoma
PAAD: Pancreatic adenocarcinoma
COAD: Colon adenocarcinoma
TMA: Tissue microarray
H&E-staining: Haematoxylin & eosin staining
IHC: Immunohistochemistry
qPCR: Quantitative real-time polymerase chain reaction
WB: Western blotting
Co-IP: Co-immunoprecipitation
IP-MS: (Immunoprecipitation - mass spectrometry)
IF: Immunofluorescence
CHX: Cycloheximide
CQ: Chloroquine
m6A: N6-Methyladenosine
