# Supplementary material for: HSP90-dependent PUS7 overexpression facilitates the metastasis of colorectal cancer cells by regulating LASP1 abundance
Source: J Exp Clin Cancer Res. 2021 May 14;40:170. doi: 10.1186/s13046-021-01951-5 (PMC8120699; doi:10.1186/s13046-021-01951-5)
Supplement: Supplementary file 1 — Additional file 1: Supplementary Table 1. Primer sequences used in the study. Supplementary Table 2. List of genes differentially expressed in DLD1-PUS7-KD versus DLD1-control cells using RNA-Seq. Supplementary Table 3. List of proteins differentially expressed in DLD1-PUS7-KD versus DLD1-control cells using Proteome-profiling. Supplementary Table 4. List of proteins potentially interacted with PUS7 and IgG in DLD1-PUS7-OE cells using Immunoprecipitation-mass spectrometry. Supplementary Table 5. Description of the patient samples acquired from clinical datasets of Xijing Hospital of Digestive Diseases [file 13046_2021_1951_MOESM1_ESM.docx]

Supplementary Table 1: Primer sequences used in the study.

| Name | 5'-3' |
| --- | --- |
| HSP90AA1-F | GATGATGAGCAGTACGCTTGG |
| HSP90AA1-R | CCTCCAAGTACTCAGTTTGGTCTTC |
| PUS7-F | ATTTGGAACCACAGCTGTCCCTA |
| PUS7-R | GCAGCAGTTGGGTCTTTGGTC |
| LASP1-F | gCAAgATgACACTgAACATgAAg |
| LASP1-R | CTgAAACCTTTgCCCTTgTTC |

Supplementary Table 2: List of genes differentially expressed in DLD1-PUS7-KD versus DLD1-control cells using RNA-Seq

| Gene_id | log2FoldChange | padj | Description |
| --- | --- | --- | --- |
| A1BG | -1.511972549 | 0.22896038 | alpha-1-B glycoprotein |
| A1CF | #NAME? | 0.00050206 | APOBEC1 complementation factor |
| A2M | -3.606462301 | 6.40E-24 | alpha-2-macroglobulin |
| ABCA13 | -1.006882149 | 0.17526842 | ATP binding cassette subfamily A member 13 |
| ABCC9 | 1.625899227 | 0.08047323 | ATP binding cassette subfamily C member 9 |
| ACOT12 | -3.63504675 | 0.29749576 | acyl-CoA thioesterase 12 |
| ACSM2B | #NAME? | 0.17507688 | acyl-CoA synthetase medium chain family member 2B |
| ADH1A | -3.642127766 | 0.01065447 | alcohol dehydrogenase 1A (class I), alpha polypeptide |
| ADH1B | -6.216927403 | 5.02E-11 | alcohol dehydrogenase 1B (class I), beta polypeptide |
| ADH6 | -3.988445383 | 0.07025486 | alcohol dehydrogenase 6 (class V) |
| AGT | -5.148546027 | 5.53E-13 | angiotensinogen |
| AGXT | -1.374817066 | 0.16156058 | alanine--glyoxylate and serine--pyruvate aminotransferase |
| AHSG | -5.102194967 | 2.31E-17 | alpha 2-HS glycoprotein |
| ALB | -7.491014156 | 9.35E-131 | albumin |
| ALDH1A1 | -2.238964588 | 0.04870036 | aldehyde dehydrogenase 1 family member A1 |
| ALDH3B2 | 1.278361691 | 0.28962146 | aldehyde dehydrogenase 3 family member B2 |
| ALDOB | #NAME? | 6.90E-11 | aldolase, fructose-bisphosphate B |
| AMBP | -5.791253469 | 1.44E-07 | alpha-1-microglobulin/bikunin precursor |
| ANGPTL3 | -2.388769576 | 0.02590012 | angiopoietin like 3 |
| ANKRD33B | 1.212266065 | 0.00084289 | ankyrin repeat domain 33B |
| APBB1 | 1.00724159 | 0.11978008 | amyloid beta precursor protein binding family B member 1 |
| APCS | #NAME? | 0.03958117 | amyloid P component, serum |
| APOA1 | -6.648906169 | 6.72E-14 | apolipoprotein A1 |
| APOA2 | #NAME? | 3.56E-19 | apolipoprotein A2 |
| APOA5 | #NAME? | 0.2249605 | apolipoprotein A5 |
| APOB | -10.2182715 | 9.52E-81 | apolipoprotein B |
| APOC2 | #NAME? | 0.00280715 | apolipoprotein C2 |
| APOC3 | #NAME? | 0.00101678 | apolipoprotein C3 |
| APOE | -1.940688608 | 8.85E-05 | apolipoprotein E |
| APOH | -5.914921121 | 4.98E-18 | apolipoprotein H |
| AQP9 | -4.74493683 | 0.00667128 | aquaporin 9 |
| ARG1 | -4.171572393 | 0.00076706 | arginase 1 |
| AZGP1 | #NAME? | 0.08566978 | alpha-2-glycoprotein 1, zinc-binding |
| BCL3 | 1.015444897 | 1.38E-17 | B cell CLL/lymphoma 3 |
| BHMT | -3.506675673 | 0.03312098 | betaine--homocysteine S-methyltransferase |
| BIRC3 | 1.888472955 | 1.62E-100 | baculoviral IAP repeat containing 3 |
| BNIP3 | -2.282489502 | 0.08260077 | BCL2 interacting protein 3 |
| BTG4 | 4.937311229 | 0.0025487 | BTG anti-proliferation factor 4 |
| C1QC | #NAME? | 0.30028903 | complement C1q C chain |
| C1S | -1.836690516 | 4.08E-05 | complement C1s |
| C3 | -1.715704809 | 2.12E-15 | complement C3 |
| C4BPA | #NAME? | 2.34E-06 | complement component 4 binding protein alpha |
| C6 | -4.342083256 | 0.01715627 | complement C6 |
| C6orf222 | 2.795108122 | 7.95E-33 | chromosome 6 open reading frame 222 |
| C8B | #NAME? | 0.22574957 | complement C8 beta chain |
| C8orf46 | 1.575276787 | 0.12052336 | chromosome 8 open reading frame 46 |
| C9 | #NAME? | 0.02048856 | complement C9 |
| CACNG8 | 2.893984839 | 1.59E-06 | calcium voltage-gated channel auxiliary subunit gamma 8 |
| CCDC170 | 1.31060218 | 0.14670349 | coiled-coil domain containing 170 |
| CCDC181 | -1.124825444 | 0.19059385 | coiled-coil domain containing 181 |
| CCL2 | Inf | 0.07662337 | C-C motif chemokine ligand 2 |
| CCL20 | 8.164565136 | 7.98E-139 | C-C motif chemokine ligand 20 |
| CCL22 | 1.455374431 | 0.30366695 | C-C motif chemokine ligand 22 |
| CCND2 | 1.111440528 | 0.1257967 | cyclin D2 |
| CD69 | 3.394085726 | 0.31541908 | CD69 molecule |
| CD74 | 1.430114314 | 0.23338109 | CD74 molecule |
| CD83 | 1.481670324 | 4.64E-44 | CD83 molecule |
| CD84 | 1.141172765 | 0.05817996 | CD84 molecule |
| CES1 | #NAME? | 4.98E-06 | carboxylesterase 1 |
| CFB | -2.868463213 | 1.38E-05 | complement factor B |
| CFH | -4.506935333 | 3.23E-08 | complement factor H |
| CFHR1 | #NAME? | 0.01685453 | complement factor H related 1 |
| CFHR2 | #NAME? | 0.09885654 | complement factor H related 2 |
| CFHR3 | #NAME? | 0.1319578 | complement factor H related 3 |
| CFI | #NAME? | 0.13574121 | complement factor I |
| CGNL1 | 1.044913836 | 0.00058273 | cingulin like 1 |
| CHST6 | 2.111599696 | 0.20468437 | carbohydrate sulfotransferase 6 |
| CIITA | 2.2073175 | 0.00039995 | class II major histocompatibility complex transactivator |
| CNTNAP1 | 1.559386311 | 4.16E-24 | contactin associated protein 1 |
| COL10A1 | -1.226799154 | 0.03489086 | collagen type X alpha 1 chain |
| COL1A2 | #NAME? | 0.29413124 | collagen type I alpha 2 chain |
| COL3A1 | -3.366199276 | 0.04150632 | collagen type III alpha 1 chain |
| COL9A2 | 5.020959176 | 0.00133342 | collagen type IX alpha 2 chain |
| COLCA2 | 1.026268362 | 0.00241213 | colorectal cancer associated 2 |
| CP | -2.110939059 | 0.00393965 | ceruloplasmin |
| CPM | -1.074243825 | 0.29792184 | carboxypeptidase M |
| CPS1 | -2.984527039 | 5.68E-21 | carbamoyl-phosphate synthase 1 |
| CREB3L3 | #NAME? | 0.22625994 | cAMP responsive element binding protein 3 like 3 |
| CSF1 | 1.836123899 | 9.21E-89 | colony stimulating factor 1 |
| CSF2 | 2.026288187 | 0.03863428 | colony stimulating factor 2 |
| CSMD3 | -1.037505204 | 0.02256974 | CUB and Sushi multiple domains 3 |
| CTSS | 1.811765615 | 0.04417045 | cathepsin S |
| CX3CL1 | 1.381481797 | 2.57E-29 | C-X3-C motif chemokine ligand 1 |
| CXCL1 | 5.218291322 | 4.65E-166 | C-X-C motif chemokine ligand 1 |
| CXCL10 | Inf | 0.07513261 | C-X-C motif chemokine ligand 10 |
| CXCL11 | 3.82561298 | 0.0105959 | C-X-C motif chemokine ligand 11 |
| CXCL12 | #NAME? | 0.22574957 | C-X-C motif chemokine ligand 12 |
| CXCL2 | 3.254743478 | 2.13E-68 | C-X-C motif chemokine ligand 2 |
| CXCL3 | 3.544834672 | 5.15E-125 | C-X-C motif chemokine ligand 3 |
| CXCL5 | 2.821482924 | 1.78E-297 | C-X-C motif chemokine ligand 5 |
| CXCL8 | 5.011115523 | 4.68E-67 | C-X-C motif chemokine ligand 8 |
| CXCR2 | 1.527870952 | 0.00369017 | C-X-C motif chemokine receptor 2 |
| CYP2A6 | -3.85196198 | 0.00343845 | cytochrome P450 family 2 subfamily A member 6 |
| CYP2B6 | -3.789296516 | 0.01481697 | cytochrome P450 family 2 subfamily B member 6 |
| CYP2C8 | -1.560095273 | 0.21527602 | cytochrome P450 family 2 subfamily C member 8 |
| CYP2C9 | -2.748923975 | 0.00110683 | cytochrome P450 family 2 subfamily C member 9 |
| CYP2E1 | -6.589101289 | 3.51E-08 | cytochrome P450 family 2 subfamily E member 1 |
| CYP3A4 | #NAME? | 1.05E-12 | cytochrome P450 family 3 subfamily A member 4 |
| CYP3A7 | -3.321537218 | 0.0710079 | cytochrome P450 family 3 subfamily A member 7 |
| CYP4A11 | -2.782118777 | 0.0298556 | cytochrome P450 family 4 subfamily A member 11 |
| CYP4F3 | -1.370024545 | 0.0002991 | cytochrome P450 family 4 subfamily F member 3 |
| CYP8B1 | #NAME? | 0.07434689 | cytochrome P450 family 8 subfamily B member 1 |
| CYTH4 | 2.239434945 | 0.09973447 | cytohesin 4 |
| DACH1 | 2.285595651 | 0.09885654 | dachshund family transcription factor 1 |
| DHRS3 | 1.141478866 | 0.00712606 | dehydrogenase/reductase 3 |
| DHRS9 | 1.76284072 | 0.03851233 | dehydrogenase/reductase 9 |
| DIO1 | -1.666126162 | 0.16662535 | iodothyronine deiodinase 1 |
| DOCK2 | 2.114464037 | 0.18058798 | dedicator of cytokinesis 2 |
| DRGX | 4.035324191 | 0.11126901 | dorsal root ganglia homeobox |
| ENHO | 1.282663906 | 0.07944764 | energy homeostasis associated |
| F2 | -1.742668423 | 0.04325485 | coagulation factor II, thrombin |
| F5 | -2.938448281 | 5.60E-05 | coagulation factor V |
| F9 | #NAME? | 0.13207032 | coagulation factor IX |
| FABP1 | #NAME? | 0.00380933 | fatty acid binding protein 1 |
| FAM167A | 1.590556176 | 0.00036812 | family with sequence similarity 167 member A |
| FCN1 | Inf | 0.18543656 | ficolin 1 |
| FDCSP | Inf | 0.18345981 | follicular dendritic cell secreted protein |
| FGA | -8.172090838 | 2.13E-26 | fibrinogen alpha chain |
| FGB | #NAME? | 9.96E-25 | fibrinogen beta chain |
| FGF20 | 1.304600738 | 0.0007865 | fibroblast growth factor 20 |
| FGG | -6.16780073 | 2.68E-19 | fibrinogen gamma chain |
| FGL1 | #NAME? | 7.69E-07 | fibrinogen like 1 |
| FMO3 | -3.613781613 | 0.30040482 | flavin containing monooxygenase 3 |
| G6PC | -4.154574563 | 0.03967367 | glucose-6-phosphatase catalytic subunit |
| GC | #NAME? | 2.10E-20 | GC, vitamin D binding protein |
| GCNT4 | 1.623259146 | 0.24762527 | glucosaminyl (N-acetyl) transferase 4, core 2 |
| GCNT7 | 2.246761139 | 0.27658553 | glucosaminyl (N-acetyl) transferase family member 7 |
| GFPT2 | 1.167252999 | 2.90E-09 | glutamine-fructose-6-phosphate transaminase 2 |
| GJB6 | 1.122844426 | 0.00356535 | gap junction protein beta 6 |
| GNLY | 3.124468012 | 0.17028277 | granulysin |
| GOLGA8B | -1.216256595 | 0.06099818 | golgin A8 family member B |
| GPR15 | 3.038289445 | 1.37E-05 | G protein-coupled receptor 15 |
| GPR37L1 | 3.257775317 | 3.31E-23 | G protein-coupled receptor 37 like 1 |
| GRIK4 | 1.510795043 | 3.21E-05 | glutamate ionotropic receptor kainate type subunit 4 |
| GSTA1 | #NAME? | 0.29792184 | glutathione S-transferase alpha 1 |
| HABP2 | -3.46501754 | 0.04806562 | hyaluronan binding protein 2 |
| HAO1 | #NAME? | 0.13424808 | hydroxyacid oxidase 1 |
| HBB | #NAME? | 0.22220758 | hemoglobin subunit beta |
| HMGCS2 | #NAME? | 1.77E-06 | 3-hydroxy-3-methylglutaryl-CoA synthase 2 |
| HP | #NAME? | 7.09E-42 | haptoglobin |
| HPCA | 1.475656464 | 0.23400476 | hippocalcin |
| HRG | -4.334555513 | 0.00042152 | histidine rich glycoprotein |
| ICAM1 | 1.236417576 | 2.67E-53 | intercellular adhesion molecule 1 |
| IGFBP1 | #NAME? | 1.21E-05 | insulin like growth factor binding protein 1 |
| IKZF1 | 4.068751782 | 0.08292638 | IKAROS family zinc finger 1 |
| IKZF3 | 1.835828077 | 3.43E-12 | IKAROS family zinc finger 3 |
| IL17C | 1.929842671 | 4.67E-06 | interleukin 17C |
| IL18RAP | Inf | 0.2488029 | interleukin 18 receptor accessory protein |
| IL2RG | 1.667195093 | 2.61E-06 | interleukin 2 receptor subunit gamma |
| IL32 | 3.471214428 | 3.05E-33 | interleukin 32 |
| IRAK2 | 1.100932318 | 7.66E-38 | interleukin 1 receptor associated kinase 2 |
| ITGA4 | 3.784677204 | 0.24221027 | integrin subunit alpha 4 |
| ITGAL | 2.267660187 | 0.14809884 | integrin subunit alpha L |
| ITGAM | 3.566884807 | 7.15E-09 | integrin subunit alpha M |
| ITGB8 | 1.012523247 | 3.92E-26 | integrin subunit beta 8 |
| ITIH1 | #NAME? | 5.02E-06 | inter-alpha-trypsin inhibitor heavy chain 1 |
| ITIH2 | -2.279573084 | 6.23E-08 | inter-alpha-trypsin inhibitor heavy chain 2 |
| ITIH3 | -5.944215048 | 5.26E-06 | inter-alpha-trypsin inhibitor heavy chain 3 |
| KCNN4 | -1.640246249 | 0.26424259 | potassium calcium-activated channel subfamily N member 4 |
| KNG1 | #NAME? | 3.99E-09 | kininogen 1 |
| KRT12 | 3.333391845 | 1.77E-06 | keratin 12 |
| KRT2 | 2.660655088 | 0.00024565 | keratin 2 |
| KRT73 | 2.519341672 | 0.22089991 | keratin 73 |
| KRTAP3-1 | 1.066113348 | 0.03723385 | keratin associated protein 3-1 |
| LAMA2 | 1.228804186 | 2.55E-17 | laminin subunit alpha 2 |
| LCN2 | 2.433918102 | 2.10E-58 | lipocalin 2 |
| LCP1 | 1.008487939 | 0.08316664 | lymphocyte cytosolic protein 1 |
| LMO7DN | -1.163799722 | 0.28303476 | LMO7 downstream neighbor |
| LOC102723996 | 1.070950887 | 3.21E-11 | ICOS ligand |
| LOC105369246 | Inf | 0.18345981 | uncharacterized LOC105369246 |
| LOC105376526 | 1.107401465 | 0.00944729 | uncharacterized LOC105376526 |
| LOC105377622 | -1.042219946 | 0.10221037 | uncharacterized LOC105377622 |
| LOC107984125 | 1.077678674 | 0.28051143 | lipoxygenase homology domain-containing protein 1-like |
| LOC107984862 | 1.41186815 | 0.00056332 | uncharacterized LOC107984862 |
| LRG1 | 1.16594091 | 1.63E-16 | leucine rich alpha-2-glycoprotein 1 |
| LRP2 | 3.594027826 | 0.24120951 | LDL receptor related protein 2 |
| LRRK2 | Inf | 0.24538559 | leucine rich repeat kinase 2 |
| LTB | 6.032223524 | 4.64E-44 | lymphotoxin beta |
| LYZ | 2.354708233 | 0.00031954 | lysozyme |
| MAP3K8 | 1.193301504 | 1.69E-18 | mitogen-activated protein kinase kinase kinase 8 |
| MAPK10 | -1.016190317 | 0.00267351 | mitogen-activated protein kinase 10 |
| MASP1 | #NAME? | 0.07476642 | mannan binding lectin serine peptidase 1 |
| MAT1A | -5.29296064 | 3.74E-05 | methionine adenosyltransferase 1A |
| MTTP | -2.081781214 | 0.20980409 | microsomal triglyceride transfer protein |
| MYO3B | -1.728633838 | 0.17181432 | myosin IIIB |
| NECAB1 | 3.357442078 | 2.98E-05 | N-terminal EF-hand calcium binding protein 1 |
| NEURL3 | 1.64904995 | 0.00013629 | neuralized E3 ubiquitin protein ligase 3 |
| NFAM1 | 3.229728795 | 0.00010355 | NFAT activating protein with ITAM motif 1 |
| NFKB2 | 1.395513033 | 8.43E-141 | nuclear factor kappa B subunit 2 |
| NFKBIA | 1.674854002 | 5.69E-87 | NFKB inhibitor alpha |
| NFKBIE | 1.513449488 | 1.09E-96 | NFKB inhibitor epsilon |
| NFKBIZ | 1.1318323 | 1.49E-84 | NFKB inhibitor zeta |
| NID1 | -2.911312548 | 0.05323232 | nidogen 1 |
| NOX1 | 1.508681914 | 3.13E-13 | NADPH oxidase 1 |
| NPHS1 | 2.154964115 | 3.79E-05 | NPHS1, nephrin |
| OLR1 | 2.015332628 | 4.33E-117 | oxidized low density lipoprotein receptor 1 |
| OMP | -1.179526111 | 0.16244025 | olfactory marker protein |
| ORM1 | #NAME? | 9.82E-19 | orosomucoid 1 |
| ORM2 | -3.743333285 | 0.2407595 | orosomucoid 2 |
| PCK1 | -3.00311543 | 0.16706449 | phosphoenolpyruvate carboxykinase 1 |
| PDE3A | -1.372607498 | 0.19822728 | phosphodiesterase 3A |
| PDE8B | -1.05276158 | 0.07318927 | phosphodiesterase 8B |
| PDZK1IP1 | 2.13148756 | 9.88E-10 | PDZK1 interacting protein 1 |
| PEG10 | -3.511371413 | 0.00031038 | paternally expressed 10 |
| PEG3 | #NAME? | 0.00577584 | paternally expressed 3 |
| PGLYRP4 | Inf | 0.18345981 | peptidoglycan recognition protein 4 |
| PHACTR3 | 1.07103474 | 0.00127229 | phosphatase and actin regulator 3 |
| PHLDB2 | 1.003118981 | 2.03E-36 | pleckstrin homology like domain family B member 2 |
| PI3 | 1.87150304 | 3.40E-06 | peptidase inhibitor 3 |
| PIK3AP1 | 1.464725442 | 1.83E-19 | phosphoinositide-3-kinase adaptor protein 1 |
| PLA2G4C | 1.186465475 | 0.17552433 | phospholipase A2 group IVC |
| PLEK | 2.598879856 | 0.17878747 | pleckstrin |
| PLEKHS1 | 2.013943909 | 0.09782715 | pleckstrin homology domain containing S1 |
| PLG | #NAME? | 5.73E-13 | plasminogen |
| PLIN2 | -1.138312185 | 0.08036425 | perilipin 2 |
| PPP1R3C | #NAME? | 0.17385755 | protein phosphatase 1 regulatory subunit 3C |
| PRF1 | 2.08134924 | 0.0008436 | perforin 1 |
| PRKCB | Inf | 0.18417734 | protein kinase C beta |
| PRKCG | 1.713489651 | 3.54E-08 | protein kinase C gamma |
| PRSS1 | -1.058048453 | 1.20E-14 | serine protease 1 |
| PRSS2 | -1.066027098 | 1.31E-66 | serine protease 2 |
| PRSS48 | -1.083403794 | 0.05817996 | serine protease 48 |
| PTGS2 | 1.371039732 | 3.00E-26 | prostaglandin-endoperoxide synthase 2 |
| PTPRC | 3.005102817 | 0.00138707 | protein tyrosine phosphatase, receptor type C |
| PTPRR | -1.261715961 | 1.19E-07 | protein tyrosine phosphatase, receptor type R |
| RBMS3 | 1.133181186 | 0.00807052 | RNA binding motif single stranded interacting protein 3 |
| RBP4 | -2.007619994 | 1.78E-06 | retinol binding protein 4 |
| REG3A | #NAME? | 0.29606545 | regenerating family member 3 alpha |
| RELB | 2.373543367 | 3.54E-39 | RELB proto-oncogene, NF-kB subunit |
| RELN | #NAME? | 0.00325526 | reelin |
| RIMS2 | -3.736666185 | 0.14998081 | regulating synaptic membrane exocytosis 2 |
| ROPN1L | -1.498620533 | 0.23765973 | rhophilin associated tail protein 1 like |
| RP1 | 2.204762188 | 7.85E-08 | RP1, axonemal microtubule associated |
| RPEL1 | -1.019724565 | 0.20546765 | ribulose-5-phosphate-3-epimerase like 1 |
| RUFY4 | 2.723845786 | 0.14563448 | RUN and FYVE domain containing 4 |
| RUNX3 | Inf | 0.10016648 | runt related transcription factor 3 |
| S100A9 | 2.602496383 | 0.01557346 | S100 calcium binding protein A9 |
| SAA1 | 5.583491382 | 3.29E-61 | serum amyloid A1 |
| SAA2 | 5.592189464 | 3.53E-77 | serum amyloid A2 |
| SAA4 | 4.197866988 | 0.04839527 | serum amyloid A4, constitutive |
| SDC2 | #NAME? | 0.09805279 | syndecan 2 |
| SDC4 | 1.551940997 | 1.07E-21 | syndecan 4 |
| SERPINA10 | #NAME? | 0.0976455 | serpin family A member 10 |
| SERPINA3 | -3.366105801 | 0.0001033 | serpin family A member 3 |
| SERPINA6 | #NAME? | 0.28950208 | serpin family A member 6 |
| SERPING1 | #NAME? | 2.83E-05 | serpin family G member 1 |
| SGPP2 | 1.331069485 | 8.74E-126 | sphingosine-1-phosphate phosphatase 2 |
| SHROOM4 | 1.537379682 | 0.21527602 | shroom family member 4 |
| SLC25A48 | 1.617084602 | 0.02481508 | solute carrier family 25 member 48 |
| SLC28A3 | 2.570577096 | 4.73E-08 | solute carrier family 28 member 3 |
| SLC2A2 | -2.660151059 | 0.01589309 | solute carrier family 2 member 2 |
| SLC2A6 | 1.167757868 | 1.72E-05 | solute carrier family 2 member 6 |
| SLC38A4 | -2.498667727 | 0.05222999 | solute carrier family 38 member 4 |
| SLC39A12 | 1.083366397 | 0.23757038 | solute carrier family 39 member 12 |
| SLFN12L | Inf | 0.18308016 | schlafen family member 12 like |
| SLFN5 | 4.352681236 | 0.01936378 | schlafen family member 5 |
| SNPH | 1.600855149 | 1.04E-42 | syntaphilin |
| SPINK4 | -2.149128212 | 0.00021242 | serine peptidase inhibitor, Kazal type 4 |
| SPP2 | #NAME? | 0.17507688 | secreted phosphoprotein 2 |
| ST6GAL1 | -1.548129626 | 0.02188215 | ST6 beta-galactoside alpha-2,6-sialyltransferase 1 |
| STAT5A | 1.759906116 | 1.57E-43 | signal transducer and activator of transcription 5A |
| SULT1C4 | 2.532282577 | 1.11E-10 | sulfotransferase family 1C member 4 |
| SULT2A1 | -4.586216677 | 0.04839527 | sulfotransferase family 2A member 1 |
| SYN3 | -2.233850275 | 0.20344129 | synapsin III |
| TAT | -3.026037845 | 0.00095588 | tyrosine aminotransferase |
| TDO2 | 2.113371048 | 1.78E-09 | tryptophan 2,3-dioxygenase |
| TF | -5.379254009 | 4.99E-12 | transferrin |
| TGM2 | -1.88683135 | 0.28655058 | transglutaminase 2 |
| TLE2 | 1.018985929 | 5.60E-05 | transducin like enhancer of split 2 |
| TM4SF4 | #NAME? | 0.00562699 | transmembrane 4 L six family member 4 |
| TMEM74B | 1.682585757 | 0.17778065 | transmembrane protein 74B |
| TNF | Inf | 0.00483335 | tumor necrosis factor |
| TNFAIP2 | 1.4308082 | 0.03427065 | TNF alpha induced protein 2 |
| TNFAIP3 | 3.010187303 | 0 | TNF alpha induced protein 3 |
| TNFRSF9 | 3.896887145 | 8.19E-52 | TNF receptor superfamily member 9 |
| TNIP1 | 1.040235115 | 6.36E-77 | TNFAIP3 interacting protein 1 |
| TRIM31 | 1.142722678 | 4.36E-07 | tripartite motif containing 31 |
| TRPA1 | 4.328743315 | 0.00015647 | transient receptor potential cation channel subfamily A member 1 |
| TTR | #NAME? | 0.00135697 | transthyretin |
| UBASH3A | Inf | 0.09837406 | ubiquitin associated and SH3 domain containing A |
| UBD | 3.699738081 | 5.47E-05 | ubiquitin D |
| UGT2B4 | -5.524815675 | 1.24E-06 | UDP glucuronosyltransferase family 2 member B4 |
| UNC13A | 1.50185509 | 6.72E-11 | unc-13 homolog A |
| UTS2 | 1.615922888 | 0.17238662 | urotensin 2 |
| VIL1 | -1.14545441 | 6.61E-13 | villin 1 |
| VTN | -3.160032078 | 1.59E-08 | vitronectin |
| WNT10A | 1.466476706 | 7.84E-10 | Wnt family member 10A |
| WNT2B | 1.045670157 | 0.2669192 | Wnt family member 2B |
| WNT4 | 1.121073871 | 0.00946646 | Wnt family member 4 |
| WNT5A | 1.30941547 | 4.70E-25 | Wnt family member 5A |
| ZC3H12A | 1.532526627 | 2.02E-71 | zinc finger CCCH-type containing 12A |
| ZNF595 | 1.158718597 | 0.10028016 | zinc finger protein 595 |

Supplementary Table 3: List of proteins differentially expressed in DLD1-PUS7-KD versus DLD1-control cells using Proteome-profiling

| **Gene Name** | **FC** | **P-value** | **Description** |
| --- | --- | --- | --- |
| PUS7 | 0.5715034 | 2.3613E-05 | Pseudouridylate synthase 7 homolog |
| S100P | 0.66620383 | 4.9993E-05 | Protein S100-P |
| TCP11L2 | 0.66852058 | 0.00029557 | T-complex protein 11-like protein 2 |
| LASP1 | 0.68558584 | 8.489E-08 | LIM and SH3 domain protein 1 |
| DEPDC1 | 0.7206768 | 1.864E-05 | DEP domain-containing protein 1A |
| MEX3C | 0.72096358 | 0.04488017 | RNA-binding E3 ubiquitin-protein ligase MEX3C |
| CRLF3 | 0.74194485 | 0.00682274 | Cytokine receptor-like factor 3 |
| SEPTIN6 | 0.76782557 | 1.8811E-05 | Septin-6 |
| C4A | 0.77095632 | 0.00021661 | Complement C4-A |
| PKIB | 0.7780083 | 0.00977804 | cAMP-dependent protein kinase inhibitor beta |
| RMI1 | 0.79509423 | 0.00970499 | RecQ-mediated genome instability protein 1 |
| SAMHD1 | 0.79964007 | 0.00477424 | Deoxynucleoside triphosphate triphosphohydrolase SAMHD1 |
| HIST1H1D | 0.80048005 | 0.00548753 | Histone H1.3 |
| NIBAN1 | 0.80258413 | 0.02117506 | Protein Niban 1 |
| KLHL11 | 0.80426939 | 0.04274301 | Kelch-like protein 11 |
| HIST1H1B | 0.80505415 | 6.8694E-05 | Histone H1.5 |
| PRR9 | 0.80747213 | 0.00377944 | Proline-rich protein 9 |
| HIST1H1E | 0.80831826 | 0.00705008 | Histone H1.4 |
| PCSK6 | 0.80916491 | 0.00762515 | Proprotein convertase subtilisin/kexin type 6 |
| CD2AP | 0.81268882 | 5.9757E-05 | CD2-associated protein |
| SP140L | 0.81543116 | 0.00223511 | Nuclear body protein SP140-like protein |
| NDRG1 | 0.8173834 | 8.4803E-06 | Protein NDRG1 |
| SESN2 | 0.81763102 | 0.02532651 | Sestrin-2 |
| SCAF11 | 0.82567691 | 2.4503E-05 | Protein SCAF11 |
| ZBTB14 | 0.82567691 | 0.03901732 | Zinc finger and BTB domain-containing protein 14 |
| PHC3 | 0.82840597 | 0.00035606 | Polyhomeotic-like protein 3 |
| RBM3 | 0.82982617 | 0.00257774 | RNA-binding protein 3 |
| BANF1 | 0.83150183 | 0.0021668 | Barrier-to-autointegration factor |
| CCND1 | 0.83236641 | 0.00195046 | G1/S-specific cyclin-D1 |
| JADE3 | 1.20066031 | 0.02638265 | Protein Jade-3 |
| H2AFX | 1.20227606 | 0.00691421 | Histone H2AX |
| GSKIP | 1.20301028 | 0.03444323 | GSK3B-interacting protein |
| ALG5 | 1.20787344 | 3.0904E-05 | Dolichyl-phosphate beta-glucosyltransferase |
| SPNS2 | 1.20957643 | 0.02048185 | Protein spinster homolog 2 |
| NDUFA12 | 1.21238938 | 0.00306305 | NADH dehydrogenase [ubiquinone] 1 alpha subcomplex subunit 12 |
| ZNF662 | 1.21283659 | 0.00016666 | Zinc finger protein 662 |
| SDC4 | 1.21565731 | 0.00050944 | Syndecan-4 |
| ARL2BP | 1.21565731 | 0.00643155 | ADP-ribosylation factor-like protein 2-binding protein |
| HIST2H2AC | 1.2164758 | 0.00039674 | Histone H2A type 2-C |
| FGG | 1.21975583 | 0.00362693 | Fibrinogen gamma chain |
| C9orf40 | 1.22020725 | 0.04100885 | Uncharacterized protein C9orf40 |
| GABARAPL1 | 1.22589021 | 0.00379711 | Gamma-aminobutyric acid receptor-associated protein-like 1 |
| KRT1 | 1.22597403 | 5.8683E-06 | Keratin, type II cytoskeletal 1 |
| GNG5 | 1.22836985 | 0.00014526 | Guanine nucleotide-binding protein G(I)/G(S)/G(O) subunit gamma-5 |
| SBSPON | 1.24215247 | 0.04745177 | Somatomedin-B and thrombospondin type-1 domain-containing protein |
| FAM76A | 1.24803297 | 0.03484957 | Protein FAM76A |
| SNX25 | 1.2556391 | 0.00172191 | Sorting nexin-25 |
| PHLDB2 | 1.26415094 | 0.00262998 | Pleckstrin homology-like domain family B member 2 |
| CXCL5 | 1.27052592 | 0.00099899 | C-X-C motif chemokine 5 |
| SEPTIN3 | 1.27186672 | 0.01713754 | Neuronal-specific septin-3 |
| KIF18A | 1.27358848 | 0.03923868 | Kinesin-like protein KIF18A |
| DGKZ | 1.28745711 | 0.00024322 | Diacylglycerol kinase zeta |
| HIST1H2AD | 1.28958413 | 0.00019733 | Histone H2A type 1-D |
| NDUFB7 | 1.29445507 | 0.02163001 | NADH dehydrogenase [ubiquinone] 1 beta subcomplex subunit 7 |
| KRT74 | 1.30985373 | 0.00052641 | Keratin, type II cytoskeletal 74 |
| CASP10 | 1.32288037 | 0.01777084 | Caspase-10 |
| KRT10 | 1.38189758 | 1.2438E-06 | Keratin, type I cytoskeletal 10 |
| KRT2 | 1.43309002 | 3.8909E-06 | Keratin, type II cytoskeletal 2 epidermal |
| OLR1 | 1.45239068 | 0.01383394 | Oxidized low-density lipoprotein receptor 1 |
| LCN2 | 1.79459711 | 1.5609E-06 | Neutrophil gelatinase-associated lipocalin |

Supplementary Table 4: List of proteins potentially interacted with PUS7 and IgG in DLD1-PUS7-OE cells using Immunoprecipitation-mass spectrometry

| Gene Symbol | # Unique Peptides | # Protein Groups | Description |
| --- | --- | --- | --- |
| PUS7 group | | | |
| KRT1 | 29 | 1 | Keratin, type II cytoskeletal 1 |
| KRT10 | 23 | 1 | Keratin, type I cytoskeletal 10 |
| KRT2 | 24 | 1 | Keratin, type II cytoskeletal 2 epidermal |
| KRT14 | 5 | 1 | Keratin, type I cytoskeletal 14 |
| KRT16 | 11 | 1 | Keratin, type I cytoskeletal 16 |
| KRT6B | 1 | 1 | Keratin, type II cytoskeletal 6B |
| KRT9 | 15 | 1 | Keratin, type I cytoskeletal 9 |
| KRT6A | 1 | 1 | Keratin, type II cytoskeletal 6A |
| KRT5 | 14 | 1 | Keratin, type II cytoskeletal 5 |
| KRT17 | 5 | 1 | Keratin, type I cytoskeletal 17 |
| KRT8 | 9 | 1 | Keratin, type II cytoskeletal 8 |
| KRT19 | 4 | 1 | Keratin, type I cytoskeletal 19 |
| HSPE1 | 8 | 1 | 10 kDa heat shock protein, mitochondrial |
| DSP | 16 | 1 | Desmoplakin |
| TUBB4B | 7 | 1 | Tubulin beta-4B chain |
| KRT18 | 9 | 1 | Keratin, type I cytoskeletal 18 |
| ACTB | 9 | 1 | Actin, cytoplasmic 1 |
| HSP90AA1 | 3 | 1 | Heat shock protein HSP 90-alpha |
| PSMB2 | 7 | 1 | Proteasome subunit beta type-2 |
| PRDX1 | 5 | 1 | Peroxiredoxin-1 |
| H3F3A; H3F3AP4; H3F3B | 5 | 1 | Histone H3.3 |
| HSPA8 | 4 | 1 | Heat shock cognate 71 kDa protein |
| YWHAZ | 4 | 1 | 14-3-3 protein zeta/delta |
| NME2; NME1-NME2 | 3 | 1 | Nucleoside diphosphate kinase B |
| TPI1 | 6 | 1 | Triosephosphate isomerase |
| PRDX2 | 4 | 1 | Peroxiredoxin-2 |
| NME1 | 3 | 1 | Nucleoside diphosphate kinase A |
| PFN1 | 5 | 1 | Profilin-1 |
| ANXA2 | 6 | 1 | Annexin A2 |
| LDHA | 4 | 1 | L-lactate dehydrogenase A chain |
| KRT78 | 4 | 1 | Keratin, type II cytoskeletal 78 |
| EEF1A1 | 6 | 1 | Elongation factor 1-alpha 1 |
| HSPD1 | 5 | 1 | 60 kDa heat shock protein, mitochondrial |
| YWHAQ | 3 | 1 | 14-3-3 protein theta |
| PEBP1 | 4 | 1 | Phosphatidylethanolamine-binding protein 1 |
| PSMB5 | 5 | 1 | Proteasome subunit beta type-5 |
| HSP90AB1 | 1 | 1 | Heat shock protein HSP 90-beta |
| RAB11A | 3 | 1 | Ras-related protein Rab-11A |
| LDHB | 4 | 1 | L-lactate dehydrogenase B chain |
| PSMB6 | 4 | 1 | Proteasome subunit beta type-6 |
| UBE2V1; TMEM189-UBE2V1 | 3 | 1 | Ubiquitin-conjugating enzyme E2 variant 1 |
| PKM | 4 | 1 | Pyruvate kinase PKM |
| APRT | 3 | 1 | Adenine phosphoribosyltransferase |
| HSPA5 | 4 | 1 | Endoplasmic reticulum chaperone BiP |
| IGKV2-29 | 2 | 1 | Immunoglobulin kappa variable 2-29 |
|  | 4 | 1 | Tubulin alpha-3D chain |
| PDIA6 | 3 | 1 | Protein disulfide-isomerase A6 |
| TOMM5 | 3 | 1 | Mitochondrial import receptor subunit TOM5 homolog |
| HIST1H4A; HIST1H4F; HIST1H4D; HIST1H4J; HIST2H4A; HIST2H4B; HIST1H4H; HIST1H4C; HIST4H4; HIST1H4E; HIST1H4I; HIST1H4B; HIST1H4K; HIST1H4L | 5 | 1 | Histone H4 |
| HNRNPK | 2 | 1 | Heterogeneous nuclear ribonucleoprotein K |
| PHB | 3 | 1 | Prohibitin |
| CCT3 | 3 | 1 | T-complex protein 1 subunit gamma |
| HIST2H2AC | 2 | 1 | Histone H2A type 2-C |
| RALY | 3 | 1 | RNA-binding protein Raly |
| PSMA3 | 3 | 1 | Proteasome subunit alpha type-3 |
| ENO1 | 5 | 1 | Alpha-enolase |
| VAPA | 5 | 1 | Vesicle-associated membrane protein-associated protein A |
| PSMB4 | 3 | 1 | Proteasome subunit beta type-4 |
| GPI | 4 | 1 | Glucose-6-phosphate isomerase |
| YWHAE | 2 | 1 | 14-3-3 protein epsilon |
| GSTP1 | 2 | 1 | Glutathione S-transferase P |
| UBE2NL | 2 | 1 | Putative ubiquitin-conjugating enzyme E2 N-like |
| RPS18 | 2 | 1 | 40S ribosomal protein S18 |
| ALDOA | 3 | 1 | Fructose-bisphosphate aldolase A |
| PPIA | 3 | 1 | Peptidyl-prolyl cis-trans isomerase A |
| HSPA1B; HSPA1A | 1 | 1 | Heat shock 70 kDa protein 1B |
| PRKDC | 2 | 1 | DNA-dependent protein kinase catalytic subunit |
| UBL4A | 2 | 1 | Ubiquitin-like protein 4A |
| RAB2B | 1 | 1 | Ras-related protein Rab-2B |
| HNRNPC | 4 | 1 | Heterogeneous nuclear ribonucleoproteins C1/C2 |
| CALR | 3 | 1 | Calreticulin |
| IMPDH2 | 5 | 1 | Inosine-5'-monophosphate dehydrogenase 2 |
| KPRP | 3 | 1 | Keratinocyte proline-rich protein |
| JUP | 2 | 1 | Junction plakoglobin |
| LGALS3 | 3 | 1 | Galectin-3 |
| YWHAB | 1 | 1 | 14-3-3 protein beta/alpha |
| ACAA2 | 2 | 1 | 3-ketoacyl-CoA thiolase, mitochondrial |
| PSPC1 | 3 | 1 | Paraspeckle component 1 |
| SSBP1 | 3 | 1 | Single-stranded DNA-binding protein, mitochondrial |
| PSMB8 | 3 | 1 | Proteasome subunit beta type-8 |
| CCT8 | 4 | 1 | T-complex protein 1 subunit theta |
| YWHAH | 1 | 1 | 14-3-3 protein eta |
| YWHAG | 2 | 1 | 14-3-3 protein gamma |
| PSMA6 | 2 | 1 | Proteasome subunit alpha type-6 |
| DSG1 | 3 | 1 | Desmoglein-1 |
| FARSB | 2 | 1 | Phenylalanine--tRNA ligase beta subunit |
| CCT2 | 6 | 1 | T-complex protein 1 subunit beta |
| HIST1H1D | 3 | 1 | Histone H1.3 |
| NHP2L1; SNU13 | 2 | 1 | NHP2-like protein 1 |
| KPNB1 | 2 | 1 | Importin subunit beta-1 |
| SNRPD3 | 1 | 1 | Small nuclear ribonucleoprotein Sm D3 |
| PSMB9 | 1 | 1 | Proteasome subunit beta type-9 |
| TPT1 | 3 | 1 | Translationally-controlled tumor protein |
| FLG2 | 2 | 1 | Filaggrin-2 |
| OCIAD1 | 1 | 1 | OCIA domain-containing protein 1 |
| GAPDH | 3 | 1 | Glyceraldehyde-3-phosphate dehydrogenase |
| HRNR | 2 | 1 | Hornerin |
| RAP1B | 1 | 1 | Ras-related protein Rap-1b |
| GANAB | 1 | 1 | Neutral alpha-glucosidase AB |
| GSN | 1 | 1 | Gelsolin |
| PDIA3 | 1 | 1 | Protein disulfide-isomerase A3 |
| PSMA8 | 1 | 1 | Proteasome subunit alpha-type 8 |
| EEF2 | 2 | 1 | Elongation factor 2 |
| S100A16 | 1 | 1 | Protein S100-A16 |
| GSTM3 | 1 | 1 | Glutathione S-transferase Mu 3 |
| VDAC1 | 1 | 1 | Voltage-dependent anion-selective channel protein 1 |
| FH | 1 | 1 | Fumarate hydratase, mitochondrial |
| YARS | 1 | 1 | Tyrosine--tRNA ligase, cytoplasmic |
| QARS | 2 | 1 | Glutamine--tRNA ligase |
| EIF4A1 | 1 | 1 | Eukaryotic initiation factor 4A-I |
| POR | 1 | 1 | NADPH--cytochrome P450 reductase |
| SPTBN1 | 2 | 1 | Spectrin beta chain, non-erythrocytic 1 |
| ENO3 | 1 | 1 | Beta-enolase |
| USP14 | 2 | 1 | Ubiquitin carboxyl-terminal hydrolase 14 |
| ATP5B | 1 | 1 | ATP synthase subunit beta, mitochondrial |
| HNRNPA2B1 | 1 | 1 | Heterogeneous nuclear ribonucleoproteins A2/B1 |
| VDAC2 | 1 | 1 | Voltage-dependent anion-selective channel protein 2 |
| EPRS | 1 | 1 | Bifunctional glutamate/proline--tRNA ligase |
| TLN2 | 1 | 1 | Talin-2 |
| ACAT1 | 1 | 1 | Acetyl-CoA acetyltransferase, mitochondrial |
| RAB7A | 1 | 1 | Ras-related protein Rab-7a |
| PSMA2 | 3 | 1 | Proteasome subunit alpha type-2 |
| PCBP3 | 1 | 1 | Poly(rC)-binding protein 3 |
| HLA-B | 1 | 1 | HLA class I histocompatibility antigen, B alpha chain |
| RAB33B | 1 | 1 | Ras-related protein Rab-33B |
| FKBP3 | 2 | 1 | Peptidyl-prolyl cis-trans isomerase FKBP3 |
| RPL12 | 1 | 1 | 60S ribosomal protein L12 |
| GLO1 | 1 | 1 | Lactoylglutathione lyase |
| PGAM1; LOC643576 | 2 | 1 | Phosphoglycerate mutase 1 |
| ACAA1 | 1 | 1 | 3-ketoacyl-CoA thiolase, peroxisomal |
| SYNJ2BP | 1 | 1 | Synaptojanin-2-binding protein |
| EEF1G | 2 | 1 | Elongation factor 1-gamma |
| CFL1 | 2 | 1 | Cofilin-1 |
| TRIM28 | 1 | 1 | Transcription intermediary factor 1-beta |
| PRDX5 | 1 | 1 | Peroxiredoxin-5, mitochondrial |
| TALDO1 | 2 | 1 | Transaldolase |
| RPS5 | 1 | 1 | 40S ribosomal protein S5 |
| GRPEL1 | 1 | 1 | GrpE protein homolog 1, mitochondrial |
| LYPLA2 | 1 | 1 | Acyl-protein thioesterase 2 |
| WDR1 | 1 | 1 | WD repeat-containing protein 1 |
| DCD | 1 | 1 | Dermcidin |
| CAPNS1 | 1 | 1 | Calpain small subunit 1 |
| PCNA | 1 | 1 | Proliferating cell nuclear antigen |
| PLIN3 | 1 | 1 | Perilipin-3 |
| CLIC1 | 2 | 1 | Chloride intracellular channel protein 1 |
| MCM7 | 1 | 1 | DNA replication licensing factor MCM7 |
| PSMA1 | 1 | 1 | Proteasome subunit alpha type-1 |
| BLVRB | 1 | 1 | Flavin reductase (NADPH) |
| ISG15 | 2 | 1 | Ubiquitin-like protein ISG15 |
| PSMB7 | 2 | 1 | Proteasome subunit beta type-7 |
| ITGB1 | 2 | 1 | Integrin beta-1 |
| HBB | 1 | 1 | Hemoglobin subunit beta |
| RPS3A | 1 | 1 | 40S ribosomal protein S3a |
| COX2 | 1 | 1 | Cytochrome c oxidase subunit 2 |
| RAB1A | 1 | 1 | Ras-related protein Rab-1A |
| NUTF2 | 1 | 1 | Nuclear transport factor 2 |
| RPS14 | 1 | 1 | 40S ribosomal protein S14 |
| PSMB3 | 2 | 1 | Proteasome subunit beta type-3 |
| DRAP1 | 1 | 1 | Dr1-associated corepressor |
| PSME3 | 1 | 1 | Proteasome activator complex subunit 3 |
| RPS7 | 1 | 1 | 40S ribosomal protein S7 |
| HUWE1 | 1 | 1 | E3 ubiquitin-protein ligase HUWE1 |
| LSM6 | 1 | 1 | U6 snRNA-associated Sm-like protein LSm6 |
| PRDX6 | 2 | 1 | Peroxiredoxin-6 |
| RPS19 | 1 | 1 | 40S ribosomal protein S19 |
| ACADVL | 1 | 1 | Very long-chain specific acyl-CoA dehydrogenase, mitochondrial |
| LAMA3 | 1 | 1 | Laminin subunit alpha-3 |
| TRNP1 | 1 | 1 | TMF-regulated nuclear protein 1 |
| LOR | 1 | 1 | Loricrin |
| TFRC | 1 | 1 | Transferrin receptor protein 1 |
| SLC35A4 | 1 | 1 | SLC35A4 upstream open reading frame protein |
| HPRT1 | 2 | 1 | Hypoxanthine-guanine phosphoribosyltransferase |
| HEXB | 1 | 1 | Beta-hexosaminidase subunit beta |
| RAN | 1 | 1 | GTP-binding nuclear protein Ran |
| LRPPRC | 1 | 1 | Leucine-rich PPR motif-containing protein, mitochondrial |
| PKP1 | 1 | 1 | Plakophilin-1 |
| NPC2 | 1 | 1 | NPC intracellular cholesterol transporter 2 |
| CAT | 1 | 1 | Catalase |
| DYNC1H1 | 1 | 1 | Cytoplasmic dynein 1 heavy chain 1 |
| CAP1 | 1 | 1 | Adenylyl cyclase-associated protein 1 |
| BID | 1 | 1 | BH3-interacting domain death agonist |
| C6orf108; DNPH1 | 1 | 1 | 2'-deoxynucleoside 5'-phosphate N-hydrolase 1 |
| CCT5 | 2 | 1 | T-complex protein 1 subunit epsilon |
| CTSD | 1 | 1 | Cathepsin D |
| ANXA3 | 2 | 1 | Annexin A3 |
| ACBD5 | 1 | 1 | Acyl-CoA-binding domain-containing protein 5 |
| PARK7 | 2 | 1 | Protein/nucleic acid deglycase DJ-1 |
| IDH3A | 1 | 1 | Isocitrate dehydrogenase [NAD] subunit alpha, mitochondrial |
| UBE2K | 1 | 1 | Ubiquitin-conjugating enzyme E2 K |
| PUS7 | 1 | 1 | Pseudouridylate synthase 7 homolog |
| ZSWIM8 | 1 | 1 | Zinc finger SWIM domain-containing protein 8 |
| SLC25A6 | 1 | 1 | ADP/ATP translocase 3 |
| ARHGDIA | 1 | 1 | Rho GDP-dissociation inhibitor 1 |
| ALB | 1 | 1 | Serum albumin |
| ILF3 | 1 | 1 | Interleukin enhancer-binding factor 3 |
| DSC1 | 2 | 1 | Desmocollin-1 |
| SATB1 | 1 | 1 | DNA-binding protein SATB1 |
| CKAP4 | 1 | 1 | Cytoskeleton-associated protein 4 |
| EIF5AL1 | 1 | 1 | Eukaryotic translation initiation factor 5A-1-like |
| PSMB1 | 3 | 1 | Proteasome subunit beta type-1 |
| UBE2M | 1 | 1 | NEDD8-conjugating enzyme Ubc12 |
| PGK1 | 1 | 1 | Phosphoglycerate kinase 1 |
| SLFN11 | 1 | 1 | Schlafen family member 11 |
| RARS | 1 | 1 | Arginine--tRNA ligase, cytoplasmic |
| CCDC40 | 1 | 1 | Coiled-coil domain-containing protein 40 |
| TKT | 2 | 1 | Transketolase |
| ATP5A1 | 1 | 1 | ATP synthase subunit alpha, mitochondrial |
| SPAG9 | 1 | 1 | C-Jun-amino-terminal kinase-interacting protein 4 |
| MDH2 | 1 | 1 | Malate dehydrogenase, mitochondrial |
| CYP2S1 | 1 | 1 | Cytochrome P450 2S1 |
| FAM49B | 1 | 1 | Protein FAM49B |
| HNRNPL | 1 | 1 | Heterogeneous nuclear ribonucleoprotein L |
| H2AFY | 1 | 1 | Core histone macro-H2A.1 |
| SSR4 | 1 | 1 | Translocon-associated protein subunit delta |
| SRP9; SRP9P1 | 1 | 1 | Signal recognition particle 9 kDa protein |
| FLG | 1 | 1 | Filaggrin |
| GCC1 | 1 | 1 | GRIP and coiled-coil domain-containing protein 1 |
| NCCRP1 | 1 | 1 | F-box only protein 50 |
| GFPT1 | 1 | 1 | Glutamine--fructose-6-phosphate aminotransferase [isomerizing] 1 |
| FLNA | 1 | 1 | Filamin-A |
| UBE2I | 1 | 1 | SUMO-conjugating enzyme UBC9 |
| UBA52 | 1 | 1 | Ubiquitin-60S ribosomal protein L40 |
| ERC1 | 1 | 1 | ELKS/Rab6-interacting/CAST family member 1 |
| C1orf68 | 1 | 1 | Skin-specific protein 32 |
| DPP3 | 1 | 1 | Dipeptidyl peptidase 3 |
| CAPZB | 1 | 1 | F-actin-capping protein subunit beta |
| SCRN3 | 1 | 1 | Secernin-3 |
| COTL1 | 1 | 1 | Coactosin-like protein |
| UBE2D3 | 1 | 1 | Ubiquitin-conjugating enzyme E2 D3 |
| LMNA | 2 | 1 | Prelamin-A/C |
| IgG group | | | |
| KRT1 | 14 | 1 | Keratin, type II cytoskeletal 1 |
| KRT2 | 10 | 1 | Keratin, type II cytoskeletal 2 epidermal |
| KRT14 | 5 | 1 | Keratin, type I cytoskeletal 14 |
| KRT10 | 10 | 1 | Keratin, type I cytoskeletal 10 |
| KRT9 | 11 | 1 | Keratin, type I cytoskeletal 9 |
| KRT6B | 5 | 1 | Keratin, type II cytoskeletal 6B |
| KRT16 | 2 | 1 | Keratin, type I cytoskeletal 16 |
| KRT5 | 3 | 1 | Keratin, type II cytoskeletal 5 |
| KRT19 | 2 | 1 | Keratin, type I cytoskeletal 19 |
| KRT8 | 1 | 1 | Keratin, type II cytoskeletal 8 |
| PSMB6 | 3 | 1 | Proteasome subunit beta type-6 |
| KRT18 | 1 | 1 | Keratin, type I cytoskeletal 18 |
| HIST1H1D | 1 | 1 | Histone H1.3 |
| UBE2NL | 1 | 1 | Putative ubiquitin-conjugating enzyme E2 N-like |
| PSMB4 | 1 | 1 | Proteasome subunit beta type-4 |
| IGKV2-40; IGKV2D-40 | 1 | 1 | Immunoglobulin kappa variable 2-40 |
| PRDX1 | 1 | 1 | Peroxiredoxin-1 |
| HIST2H2AC | 1 | 1 | Histone H2A type 2-C |
| NME1 | 1 | 1 | Nucleoside diphosphate kinase A |
|  | 1 | 1 | Tubulin alpha-3D chain |
| GAPDH | 1 | 1 | Glyceraldehyde-3-phosphate dehydrogenase |
| TUBB2A | 1 | 1 | Tubulin beta-2A chain |
| HIST1H4A; HIST1H4F; HIST1H4D; HIST1H4J; HIST2H4A; HIST2H4B; HIST1H4H; HIST1H4C; HIST4H4; HIST1H4E; HIST1H4I; HIST1H4B; HIST1H4K; HIST1H4L | 1 | 1 | Histone H4 |
| ACTB | 3 | 1 | Actin, cytoplasmic 1 |
| NRDE2 | 1 | 1 | Nuclear exosome regulator NRDE2 |
| DPF3 | 1 | 1 | Zinc finger protein DPF3 |
| H3F3A; H3F3AP4; H3F3B | 1 | 1 | Histone H3.3 |
| KANSL1L | 1 | 1 | KAT8 regulatory NSL complex subunit 1-like protein |
| CSTF1 | 1 | 1 | Cleavage stimulation factor subunit 1 |
| ZNF345 | 1 | 1 | Zinc finger protein 345 |
| YWHAZ | 1 | 1 | 14-3-3 protein zeta/delta |
| SCNM1; TNFAIP8L2-SCNM1 | 1 | 1 | Sodium channel modifier 1 |

Supplementary Table 5: Description of the patient samples acquired from clinical datasets of Xijing Hospital of Digestive Diseases

| **ID** | **Gender** | **Age** | **Tumor differentiation** | **Disease** |
| --- | --- | --- | --- | --- |
| 1 | female | 72 | moderate | COAD |
| 2 | female | 53 | moderate | COAD |
| 3 | male | 44 |  | READ |
| 4 | female | 66 | moderate | READ |
| 6 | male | 78 | moderate | READ |
| 7 | female | 55 | moderate | COAD |
| 8 | female | 36 | moderate | COAD |
| 9 | male | 88 | moderate | COAD |
| 10 | female | 80 | moderate | COAD |
| 11 | female | 53 | moderate | CRC |
| 13 | female | 50 | moderate | COAD |
| 14 | male | 67 | moderate | READ |
| 15 | male | 54 | poor | COAD |
| 16 | male | 47 | poor | COAD |
| 17 | male | 61 | moderate | READ |
| 18 | female | 46 | well | COAD |
| 19 | male | 62 | moderate | READ |
| 20 | female | 37 | moderate | COAD |
| 21 | female | 80 | moderate | COAD |
| 22 | female | 63 | moderate | COAD |
| 23 | male | 45 | well | COAD |
| 24 | female | 47 | moderate | COAD |
| 25 | male | 41 | moderate | READ |
| 26 | female | 65 | moderate | COAD |
| 27 | male | 75 | moderate | READ |
| 28 | male | 61 | moderate | READ |
| 29 | male | 67 | moderate | READ |
| 30 | male | 84 | moderate | READ |
| 31 | male | 79 | moderate | COAD |
| 32 | female | 78 | moderate | READ |
| 33 | male | 73 | moderate | READ |
| 34 | female | 38 | moderate | COAD |
| 35 | male | 82 | moderate | COAD |
| 36 | male | 76 | moderate | COAD |
| 37 | male | 48 | moderate | READ |
| 38 | male | 63 | moderate | COAD |
| 39 | male | 59 | well | READ |
| 40 | male | 43 | well | COAD |
| 41 | female | 38 | poor | READ |
| 42 | male | 49 |  | READ |
| 43 | male | 62 |  | COAD |
| 44 | male | 70 | moderate | READ |
| 45 | male | 59 | moderate | READ |
| 46 | male | 65 | moderate | COAD |
| 47 | male | 78 | moderate | COAD |
| 48 | male | 65 | moderate | READ |
| 49 | male | 70 | moderate | READ |
| 50 | male | 49 | moderate | COAD |
| 51 | female | 59 | moderate | COAD |
| 52 | female | 64 | moderate | COAD |
| 53 | male | 77 | moderate | READ |
| 54 | male | 69 | moderate | READ |
| 55 | male | 31 | moderate | COAD |
| 56 | male | 64 | moderate | READ |
| 57 | female | 82 | moderate | READ |
| 58 | female | 76 | moderate | COAD |
| 59 | male | 46 | moderate | READ |
| 60 | male | 48 |  | READ |
| 61 | male | 71 |  | READ |
| 62 | female | 75 | moderate | COAD |
| 63 | male | 84 | moderate | READ |
| 64 | female | 71 | moderate | READ |
| 65 | female | 78 | moderate | READ |
| 66 | female | 72 | moderate | COAD |
| 67 | male | 73 | moderate | READ |
| 68 | female | 54 | well | COAD |
